# Activation of butterfly eyespots by Distal-less is consistent with a reaction-diffusion process

**DOI:** 10.1242/dev.169367

**Published:** 2019-05-09

**Authors:** Heidi Connahs, Sham Tlili, Jelle van Creij, Tricia Y. J. Loo, Tirtha Das Banerjee, Timothy E. Saunders, Antónia Monteiro

**Affiliations:** 1 Department of Biological Sciences, National University of Singapore, Singapore 117558; 2 Mechanobiology Institute, National University of Singapore, Singapore 117411; 3 Institute of Molecular and Cell Biology, A*Star, Proteos, Singapore 138673; 4 Yale-NUS College, Singapore 138527

**Keywords:** CRISPR-Cas9, Butterfly eyespots, Distal-less, Gray–Scott reaction-diffusion model, Morphogens

## Abstract

Eyespots on the wings of nymphalid butterflies represent colorful examples of pattern formation, yet the developmental origins and mechanisms underlying eyespot center differentiation are still poorly understood. Using CRISPR-Cas9 we re-examine the function of Distal-less (Dll) as an activator or repressor of eyespots, a topic that remains controversial. We show that the phenotypic outcome of CRISPR mutations depends upon which specific exon is targeted. In *Bicyclus anynana*, exon 2 mutations are associated with both missing and ectopic eyespots, and also exon skipping. Exon 3 mutations, which do not lead to exon skipping, produce only null phenotypes, including missing eyespots, lighter wing coloration and loss of scales. Reaction-diffusion modeling of Dll function, using Wnt and Dpp as candidate morphogens, accurately replicates these complex crispant phenotypes. These results provide new insight into the function of Dll as a potential activator of eyespot development, scale growth and melanization, and suggest that the tuning of Dll expression levels can generate a diversity of eyespot phenotypes, including their appearance on the wing.

This article has an associated ‘The people behind the papers’ interview.

## Abstract (precis)

**Highlighted Article:** CRISPR-generated mutants and theoretical modeling show that Dll is a crucial activator of butterfly wing eyespot center differentiation.

## INTRODUCTION

The genetic and developmental origins of the bullseye color patterns on the wings of nymphalid butterflies are still poorly understood. Eyespots originated in ancestors of this butterfly lineage, around 90 million years ago (Monteiro, 2015; Oliver et al., 2012, 2014), most likely to function as targets for deflecting predators away from the butterfly's vulnerable body (Monteiro, 2015; Olofsson et al., 2010; Prudic et al., 2015). Eyespots may have originated via the co-option of a network of pre-wired genes because several of the genes associated with eyespots gained their novel expression domain concurrently with the origin of eyespots (Oliver et al., 2012). Some of these genes have since lost their expression in eyespots, without affecting eyespot development, suggesting that they did not play a functional role in eyespot development from the beginning (Oliver et al., 2012). Yet, one of the genes, *Distal-less* (*Dll*), has remained associated with eyespots in most nymphalid species examined so far, suggesting that it may have played a functional role in eyespot origins (Oliver et al., 2012; Shirai et al., 2012).

The function of *Dll* in eyespot development was initially investigated in *B. anynana* using transgenic overexpression, RNA interference (RNAi) and ectopic expression tools (Monteiro et al., 2013). Overexpression of *Dll* in *B. anynana* led to the appearance of small additional eyespots on the wing as well as larger eyespots, whereas *Dll* downregulation produced smaller eyespots, strongly implicating *Dll* as an activator of eyespot development (Monteiro et al., 2013). However, a recent study using CRISPR-Cas9 to knock out *Dll* function in the painted lady butterfly *Vanessa cardui* contradicted these findings. Zhang and Reed (2016) found that using two guides to disrupt exon 2 in *Dll* led to the appearance not only of distally extended eyespots but also of ectopic eyespots developing in novel locations on the wing. These observations led to the conclusion that *Dll* represses eyespot development. In addition, these researchers also showed that targeting the same exon in another butterfly, *Junonia coenia*, produced darker wing pigmentation, whereas the exact same phenotype was obtained via ectopic expression of *Dll* in the wings of *B. anynana* (Monteiro et al., 2013) and in the wings of *J. orithya*, a close relative of *J. coenia* (Dhungel et al., 2016). One possibility for the discrepancies seen across species is that *Dll* has precisely opposite functions in the different butterfly species. Another possibility, which we believed more likely, is that the outcomes of genome editing may depend on the particular site that is targeted in the genome to disrupt the gene's function.

**Fig. 1.**
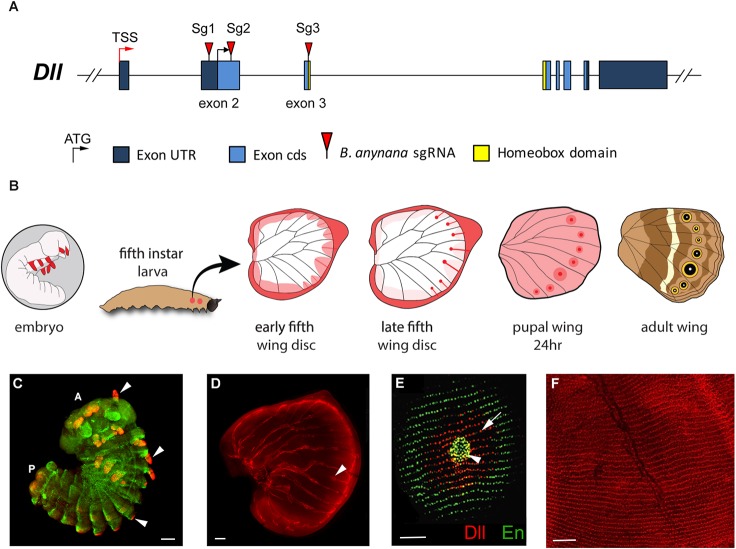
**Expression of Distal-less in embryos,** **and** **larval and pupal wings.** (A) *Dll* gene structure indicating the exons targeted by guide RNAs in this work (red triangles). TSS, transcription start site. (B) Summary diagram of relevant expression patterns of Dll in embryo limbs and different stages of fifth instar and pupal wings. Dll is represented as a gradient of pink to red illustrating weaker to stronger expression, respectively, with highest expression in the wing margin and also fingers terminating in an eyespot center. This temporal expression pattern of Dll in the larval and pupal wings has been replicated in numerous studies (Brakefield et al., 1996; Monteiro et al., 2013; Oliver et al., 2013, 2014; Reed and Serfas, 2004). (C-F) Fluorescent immunostainings of Dll (red) and Engrailed (En, green). In pupal wings, Dll is expressed in all scale-building cells (at low levels) and at higher levels in the scale cells that will become the black disc of an eyespot. (C) Dll is expressed in antennae, thoracic legs, and abdominal prolegs of embryos (arrowheads), A, anterior; P, posterior. En is also expressed in embryos. Photo credit: Xiaoling Tong (Southwest University, Chongqing, China). (D) Dll is expressed in eyespot centers (arrowhead) and along the wing margin in late larval wings. (E) Dll is expressed in eyespot centers (arrowhead) and in black scale cells of pupal wings (arrow). En is expressed in the eyespot center and area of the gold ring. (F) Dll expression in rows of scales across the entire surface of a 24-h pupal wing. Scale bars: 100 µm in C,D; 50 µm in E,F.

In order to clarify the function of *Dll* in *B. anynana*, we separately targeted both exon 2 (using single guide RNAs Sg1 and Sg2) and exon 3 (using Sg3, which targets the homeobox) in the same species, *B. anynana* (Fig. 1A). While screening potential crispants (mutants with CRISPR-induced phenotypes), we paid special attention to areas in which *Dll* expression was previously detected in this species. These areas included the antennae, thoracic and abdominal legs (Saenko et al., 2008; Tong et al., 2014), eyespot centers (Brakefield et al., 1996; Brunetti et al., 2001), scale-building cells across most of the wing (at low levels) and those of the eyespot black discs (at high levels) (Brunetti et al., 2001; Monteiro et al., 2007) and the wing margin including the parafocal elements (Brakefield et al., 1996) (Fig. 1B-F).

To explore further the role of Dll in eyespot development, we complemented our functional experiments with a theoretical modeling approach. Reaction-diffusion modeling has been used to simulate a variety of complex patterns in nature, such as color patterning in vertebrates, digit specification in mice, and the distal fin elements in catsharks (Kondo and Miura, 2010; Onimaru et al., 2016; Raspopovic et al., 2014). Reaction-diffusion models have also been used to model eyespot center differentiation during the larval stage (Nijhout, 1990; Sekimura et al., 2015), as well as the later process of ring differentiation during the pupal stage (Dilão and Sainhas, 2004). However, such models have not been tested under controlled experimental perturbation, e.g. by altering the local distribution of some of the required components. Further, specific molecular components involved in eyespot center differentiation remain largely unknown. Using spatial-temporal expression data in larval wings for Dll, Armadillo (Arm; a Wnt signal transducer), and *decapentaplegic* (*dpp*), we modeled a putative network incorporating Wnt and Dpp signaling along with Dll that leads to eyespot center differentiation. Our model can reproduce both wild-type eyespots as well as CRISPR eyespot phenotypes by simply perturbing effective Dll levels. We integrate spatial, temporal and molecular information within our model to provide further insight into the functional role of Dll during eyespot center differentiation.

Our main findings show that targeting different exons in *B. anynana* can lead to the development of opposite phenotypes. Whereas targeting both exons resulted in loss of eyespots, in some cases targeting exon 2 led to ectopic eyespots. The challenges of working on a non-model organism prevented us from uncovering the precise mechanism producing these gain-of-function phenotypes; however, our functional and theoretical approach allowed us to propose a new model describing Dll function in eyespot center differentiation. Our model defines Dll as a required activator of eyespots for which expression levels determine eyespot number and size. Our experiments and modeling also support a potential functional role for the morphogenetic Wnt ligands and Dpp in eyespot center differentiation, center size, and positioning, although we cannot exclude other possible morphogens playing a role.

## RESULTS

We injected embryos with single guide RNAs (sgRNAs) targeting either exon 2 or exon 3 of *Dll*, after confirming that these guides worked *in vitro*. To confirm guide RNA efficiency *in vitro*, we purified genomic amplicons of *Dll*, containing either exon 2 or exon 3, and treated them with the respective guide RNAs and with Cas9 protein. The resulting products, when run on a gel, showed two bands of the predicted sizes for Sg1 and Sg3 and a faint band for Sg2 (Fig. S1), confirming that the CRISPR-Cas9 system was introducing double-strand breaks in the targeted sequences*.*

### Dll exon 3 crispants produce loss-of-function phenotypes

**Fig. 2.**
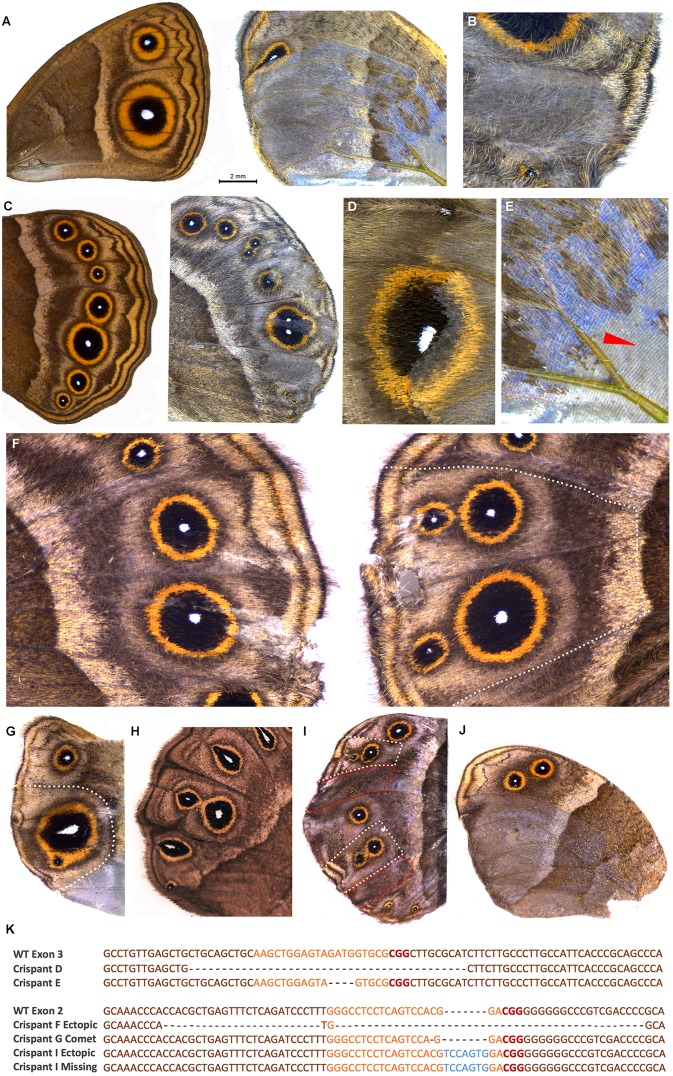
**Crispants generated by targeting exon 2 and exon 3 of Dll.** (A) Wild-type forewing of *B. anynana* (left) and exon 3 phenotype with eyespots missing in areas of lighter pigmentation and disrupted venation (right). (B) Exon 3 phenotype with missing eyespot in a patch with mutant clones. (C) Wild-type hindwing of *B. anynana* (left) and exon 3 phenotype with split eyespots and bisected eyespot centers. (D) Exon 3 phenotype showing light colored scales in mutant clones across an eyespot. (E) Exon 3 phenotype showing close-up of crispant in A with a region of missing scales as indicated by the red arrowhead. (F-J) Exon 2 mutations. (F) Wild-type (left) and crispant wing (right) of the same individual in which ectopic eyespots appeared on the distal hindwing margin after exon 2 was targeted. (G) Comet-shaped Cu1 eyespot center. (H) Example of a spontaneous comet mutant. (I) Wing with ectopic eyespots as well as missing eyespots. (J) Missing eyespots on hindwing in mosaic areas also showing lighter pigmentation. (K) Next-generation sequencing of selected crispants (exon 3 top panel and exon 2 bottom panel) identifying the most frequent indels around the target site. Orange, guide region; red, PAM sequence; blue, insertions; dashed lines, deletions. Dotted lines on exon 2 crispants in F, G and I represent wing regions carefully dissected for DNA isolation. Wing sectors for the crispant shown in I, outlined in red (missing eyespots), were pooled for DNA isolation as were wing sectors outlined in white (ectopic eyespots). For the exon 3 crispant in C the entire distal wing margin was dissected and for the crispant in D the area around the eyespot was dissected.

Embryonic injections of Sg3 targeting the *Dll* homeobox sequence on exon 3 (Fig. 1A; Table 1) led to a variety of adult phenotypes (Fig. 2; Table 2). The most striking crispants displayed complete loss of eyespots (Fig. 2A,B) followed by eyespots with significant developmental perturbations. Altered or lighter scale pigmentation appeared to correspond to the extent of the mutant clones. Depending on their location, the lighter patches of wing tissue (i.e. the presumptive *Dll* mutant clones) had remarkable effects on pattern formation. Eyespots vanished when mutant patches covered the location of the eyespot centers (Fig. 2A,B), and split eyespots emerged when the mutant tissue bisected normal eyespot centers (Fig. 2C). Some patches also had lighter gray-blue scale pigmentation, colors that result from the loss of scale pigments (Fig. 2D; Fig. S2), and lacked cover scales, or both cover and ground scales (Fig. 2E). In addition to wing pattern mutations, we observed appendage defects that would be expected from a *Dll* knockout (Chen et al., 2016; Panganiban, 2000). A number of crispants exhibited barely noticeable stumps, legs with missing tarsi (Fig. S3A) and deformed antennae with missing tips (Fig. S3B).

**Table 1.**
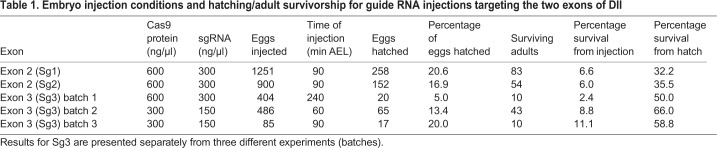
**Embryo injection conditions and hatching/adult survivorship for guide RNA injections targeting the two exons of Dll**

**Table 2.**
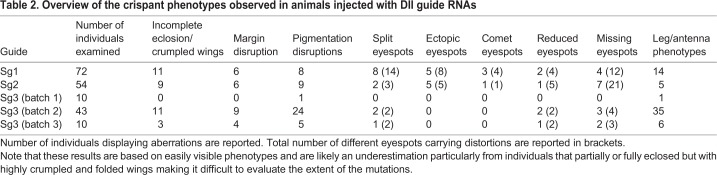
**Overview of the crispant phenotypes observed in animals injected with Dll guide RNAs**

### *Dll* exon 2 crispants produce gain- and loss-of-function phenotypes

Embryonic injections of guide RNAs targeting either the 5′UTR (Sg1) or the coding sequence (Sg2) of exon 2 led to phenotypes similar to those described above (Table 2) as well as to a remarkable new set of phenotypes, sometimes co-occurring on the same wing. These included ectopic eyespots along the proximal-distal axis of the wing (Fig. 2F) and eyespots with a tear-drop-shaped center (Fig. 2G), closely resembling a spontaneous mutant variant in *B. anynana* known as the comet phenotype (Brakefield, 1998) (Fig. 2H). Ectopic eyespots were observed regardless of whether we targeted the 5′UTR or the coding sequence of exon 2, as we injected each of these guide RNAs separately. Some butterflies displayed both ectopic and missing eyespots on the same wing (Fig. 2I). Interestingly, ectopic eyespots were never associated with changes in pigmentation, in contrast to wing tissue with missing eyespots (Fig. 2I,J), which always displayed the gray-blue pigmentation defects, highlighting the extent of the mutant clone of cells. Similar to exon 3 crispants, we also observed appendage defects, including truncated antenna and legs or fusion of antenna or proximal leg segments (Fig. S3C-E).

### Confirmation of CRISPR-Cas9 activity using next-generation sequencing

In order to confirm that the phenotypes observed were due to genetic alterations of the targeted exons, we performed next-generation amplicon sequencing of *Dll* to identify the entire range of mutations generated from Sg1, representing exon 2 mutations, and Sg3, representing exon 3 mutations. To identify mutations associated with each specific phenotype, especially in the case of exon 2 mutations that produced both ectopic as well as missing eyespots, we isolated DNA from the adult wing tissue by carefully dissecting around regions corresponding to missing, ectopic or comet eyespots, and characterized them separately (see Fig. 2F-G,I). To characterize mutations we used CRISPResso, a software pipeline for analyzing next-generation sequencing data generated from CRISPR-Cas9 experiments (Pinello et al., 2016). This analysis identified a range of mutations from each wing tissue, including deletions and insertions (Figs S4, S5, Table S1); the most frequent mutations are shown in Fig. 2K. The majority of mutations in exon 3 were large or frame-shift deletions whereas mutations induced by Sg1 were mostly non-coding (Table S2). For Sg3, we sequenced two individuals (Fig. 2C,D) and identified a range of mutations with the most frequent representing a 42 bp and 4 bp deletion, respectively (Fig. 2K; Fig. S4A). For Sg1, we sequenced three individuals (Fig. 2F,G,I). A large 72 bp deletion was observed in a crispant displaying ectopic eyespots (Fig. 2F; Fig. S4B). In contrast, relatively small indels were observed for another ectopic eyespot crispant (Fig. 2I,K; Fig. S4C), and, surprisingly, the same 7 bp insertion emerged as the most frequent mutation from wing tissue with either ectopic or missing eyespots (Fig. 2I,K). The most frequent mutation observed for the comet eyespot phenotype represented a single base pair deletion (Fig. 2G,K; Fig. S4B). Overall, CRISPResso identified only a very small proportion of mutations as disruptions to potential splice sites (0.1-0.2%). Because the link between specific mutations and the observed phenotypes was not clear, we decided to explore whether mutations that targeted each of the exons led to modifications in the way that *Dll* was transcribed.

### Targeting exon 2 induces alternative splicing

To explain the presence of ectopic eyespots following exon 2 disruptions, we examined the resulting cDNA sequences. RNA was isolated from embryos injected with Cas9 and each of the three guides, as well as from wild-type non-injected embryos. PCR amplification from cDNA using primers spanning exon 1 to exon 6 revealed that embryos injected with either Sg1 or Sg2, targeting exon 2, produced a novel product approximately 500 bp shorter than the wild-type product. Sequencing this short product revealed a deletion of 492 bp representing exon 2, suggesting that this exon had been completely spliced out. In contrast, we did not observe any alternative splicing for cDNA obtained from wild-type embryos or embryos injected with Sg3 (Fig. S6A). These experiments were replicated (*n*=4) with a pool of 50 embryos per replicate and confirmed exon skipping when using Sg1 and Sg2 (Fig. S6B).

To understand the underlying cause of this aberrant skipping of exon 2, we performed a search for predicted exonic splicing enhancers (ESEs) in this exon using ESEfinder (Cartegni et al., 2003). ESEs function as cis-elements that contain recognition sites for SR proteins (serine/arginine-rich proteins), which are involved in recruiting the splicesosome machinery (Cartegni et al., 2003). Mutations in ESEs can result in aberrant splicing and exon skipping, which can have important consequences for development and disease (Caputi et al., 2002; Zatkova et al., 2004). Our analysis revealed that the two highest scoring predicted ESE motifs for exon 2 land exactly within Sg1 and Sg2 sequences, respectively (Table S3). ESEs of exon 2 were therefore likely disrupted in the crispants leading to exon 2 skipping.

To examine whether targeting exon 2 resulted in ectopic eyespots owing to *Dll* overexpression, we performed qPCR on cDNA from embryos injected either with Sg1 or Sg3, using primers designed to amplify exon 1. The aim of this experiment was to capture all *Dll* transcripts including the alternatively spliced variants and to quantify them. The results did not reveal any significant differences in *Dll* expression after normalizing the data to the internal control gene EF1 alpha (*Dll* exon 1; *P*=0.66, *P*=0.08). Overall expression levels of *Dll* were low, with average Ct values of 29.9±0.4 (s.e.m.) (Sg1) and 30.6±0.5 (s.e.m.) (Sg3) (*n*=4). This experiment suggests that exon 2 disruptions do not affect *Dll* expression levels directly, but perhaps affect downstream, post-transcriptional processes, such as rates of protein degradation in cells. Alternatively, expression levels of *Dll* during the embryonic stage, quantified here, could differ from those occurring during the period of eyespot formation in the larval wing disc.

### Morphogenetic signals are dynamically distributed in each developing wing sector

Several of the *Dll* crispant phenotypes, including missing eyespots, suggested that this gene is involved in the process of eyespot center differentiation, which takes place during the late larval stage (Brakefield et al., 1996; Carroll et al., 1994) (Fig. 1B). However, we noticed intriguing phenotypes, such as splitting of eyespot centers within a single wing sector bordered by veins, and deformities in eyespot centers and color rings near boundaries of wild-type and mutant tissue. These phenotypes suggest that Dll might interact with spatially varying proteins and also the vein boundaries to set up eyespot centers. We hypothesized that morphogen signals might play a key role by interacting with Dll to create the eyespot center. To test this, we examined the spatial expression of two members of two candidate signaling pathways: *decapentaplegic* (*dpp*), coding for the Dpp morphogen; and Armadillo (Arm), the signal transducer of Wingless (Wnt) morphogens (Klingensmith and Nusse, 1994) in 5th instar larvae. We focused on these pathways as Wnt1 and Dpp morphogens are known to be involved in *Dll* regulation in early leg discs of *Drosophila* (Estella et al., 2008).

**Fig. 3.**
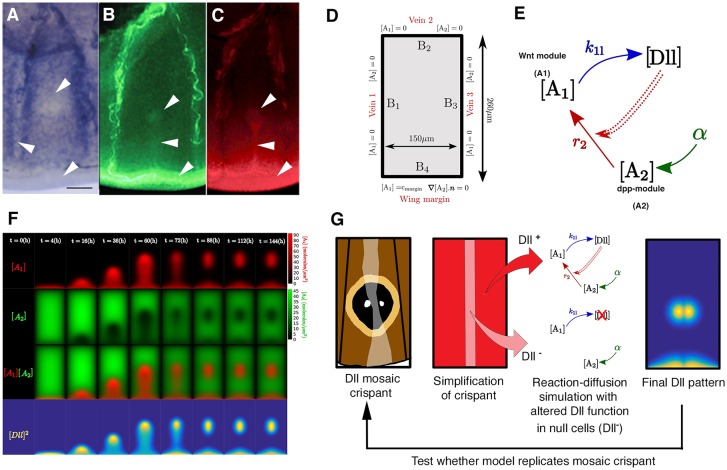
**Morphogenetic inputs and modeling of eyespot formation.** (A) In late fifth instar wing discs, *dpp* is expressed across the wing compartment but levels are lower in the eyespot centers, along the veins and along the wing margin (arrowheads). (B) Arm is located in eyespot centers, at the midline, as well as the wing margin (arrowheads) at the same time. (C) *Dll* has a similar localization pattern to Arm also in late fifth wing discs. (D) Boundary conditions and size for the wing compartment. The boundaries with veins are modeled as sinks for both *A_1_* and *A_2_*. At the wing margin, *A_1_* is imposed at a fixed concentration *c_margin_*, whereas we impose zero-flux conditions on *A_2_*. (E) Interaction network involving the activator *A_1_*, the substrate *A_2_* and *Dll*. *Dll* interacts cooperatively with itself (double dashed line) and with *A_2_* to induce *A_1_.* During the reaction [A_2_]+2[Dll]→[A_1_], *A_2_* is degraded. *A_2_* is produced uniformly throughout the sector. See also Eqns 1 and 2 for model formulation. (F) Time-lapse results of reaction-diffusion simulation of eyespot center formation. Concentration of A_1_ (first row) and A_2_ (second row) over 6 days is shown. Third row is the overlap of A_1_ and A_2_. Fourth row represents the Dll signaling incorporated within the model (see E). (G) To test our model, we represented the actual Dll mutant clones (light-colored stripe intersecting eyespot, represented as a light pink stripe with Dll-null function) in the modeled wing sector and ran the model to observe how the final generated pattern compared with the observed pattern. Scale bar: 100 µm in A.

We cloned an 810 bp fragment for *B. anynana dpp* using specific primers (Table S4) and performed *in situ* hybridization. For visualization of Arm protein we used antibodies developed against a *Drosophila* homolog (Colosimo and Tolwinski, 2006). In young fifth instar wing discs, we observed a *dpp* stripe in the middle of the wing discs, separating anterior from posterior wing compartments, as expected from work on *Drosophila* (Lecuit et al., 1996) (Fig. S7A). In later fifth instar wing discs, *dpp* was expressed across the whole wing, except along the veins and wing margin, with slightly elevated expression in regions flanking each vein, and reduced expression in the future eyespot centers as well as in the midline of each wing sector (Fig. 3A; Fig. S7B,C). At a late larval stage, *dpp* expression declined everywhere with the exception of the antero-posterior stripe (Fig. S7D). Arm, on the other hand, showed an anti-colocalized pattern with *dpp*; it was highly expressed in areas where *dpp* was missing, e.g. along the wing margin, and in the eyespot centers and in the midline (Fig. 3B; Fig. S7E,F). Below, we use the information from these dynamic gene expression patterns, as well as from *Dll*, to model eyespot center differentiation.

The expression profiles of *dpp* change relatively rapidly during the fifth instar stage of *B. anynana*, suggesting long-range kinetics consistent with diffusion (Kicheva et al., 2007) (Tables S5 and S6), but there is currently an absence of direct kinetic data for morphogens in butterfly wings. Özsu et al., however, have shown that downregulation of Wg (Wnt1) at the end of larval stages in *B. anynana* results in smaller eyespots (Özsu et al., 2017), implicating Wnt1 as a potential activator within this reaction. Meanwhile, we observe that *dpp* is excluded from the eyespot center, suggesting an interaction between *dpp* and genes localized in the eyespot center.

### Activator-substrate model with anti-colocalized morphogenetic factors can create eyespot centers

The above results suggest a model in which Dll is necessary to define eyespots and its spatial range of action is, at least in part, regulated by Dpp and Wg. To test this model, and to probe potential mechanisms of eyespot center formation, we utilized theoretical modeling to explore putative interactions between morphogens and the transcription factor Dll. Although we do not have a wide-range of genetic mutations to challenge the model (owing to difficulties with making stable genetic perturbations in butterflies), the variety and complexity of the observed eyespot patterns under *Dll* perturbation provides a powerful dataset. We incorporate the experimental observations of the previous section within an activator-substrate (Gray–Scott) reaction-diffusion model (Gierer and Meinhardt, 1972; Gray and Scott, 1984). Most previous modeling of eyespot formation has used a Gierer–Meinhardt activator-inhibitor model (Sekimura et al., 2015). A key reason for our model choice is that in the activator-substrate models, new spot centers can form by a single spot splitting into two (Koch and Meinhardt, 1994), similar to our observations in *Dll* crispants, as well as in spontaneous comet mutants of *B. anynana* (Fig. 2H; Figs S8-S10). In contrast, in the Gierer–Meinhardt activator-inhibitor model, (Sekimura et al., 2015) (supplementary Materials and Methods) new spots of the activator (i.e. eyespot centers) typically form between two existing spots. Importantly, in the activator-substrate model the morphogenetic inputs can be anti-colocalized, in contrast to their colocalization in the Gierer–Meinhardt model (Gierer and Meinhardt, 1972).

Our model incorporates three essential elements: an autocatalytic activator (A_1_) module, which likely incorporates the action of one or more Wnts; a substrate (A_2_) module that is degraded during activator production, which likely incorporates the action of Dpp; and *Dll*, which acts as an intermediary between the activator and substrate. Specifically, we included *Dll* within the network as a downstream gene activated by A_1_, which initially is expressed only along the wing margin. This is supported by the observation of colocalization of Arm and Dll (Fig. 3B,C) and by the assumption that the known activation of *Dll* by Wnt1 (via Arm) in the *Drosophila* wing margin (Neumann and Cohen, 1997) is conserved in butterflies. Further, ectopic Dll can activate endogenous *Dll* as well as *wg* in the wing and leg discs of *Drosophila* (Gorfinkiel et al., 1997). Of course, we do not know the actual interactions between Dll, Wg and Dpp in *B. anynana* wing discs, so we use the general Gray–Scott kinetics for simplicity. The substrate A_2_ (which incorporates Dpp) is uniformly produced throughout the wing compartment at a rate α, consistent with our *dpp in situ* observations (Fig. 3A; Fig. S7). We emphasize that A_1_ and A_2_ likely do not correspond to single molecules and that the interactions between components are approximations of more complex underlying kinetics. However, this model is more consistent with the above data and previously published work than other models of eyespot center formation. Owing to the lack of detailed kinetic analysis of protein dynamics in the wing disc, we cannot discount alternative mechanisms, such as direct cell-cell signaling, in formation of the eyespot centers.

In this formulation, the concentrations of A_1_ and A_2_, denoted by [*A*_1_] and [*A*_2_], are described by:
(1)
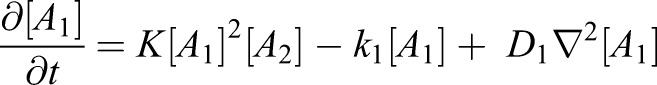


and
(2)
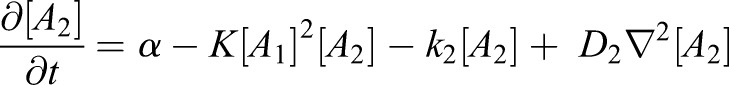


where 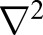 represents the two-dimensional Laplacian operator. The action of Dll is included within the non-linear reaction term (*K*[*A*_1_]^2^[*A*_2_]) (see supplementary Materials and Methods for further details). The values of the diffusion coefficients (*D_1_* and *D_2_*), the degradation rates (*k_1_* and *k_2_*) and production rate (α) are constrained by measurements in *Drosophila* (Kicheva et al., 2007). In contrast, the value of the parameter representing the interaction between Dll, A_1_ and A_2_ (*K*) is unknown (see Materials and Methods and supplementary Materials and Methods for detailed description of simulation implementation, boundary conditions, initial conditions, and parameter tables). We further fixed our parameter values to lie within the spot formation region of the phase space, where the reaction producing A_1_ degrades A_2_ at the same rate (Chen and Ward, 2011; Rasmussen et al., 1996) (Figs S9, S11, supplementary Materials and Methods).

This reaction network produced a broad patch of activator (A_1_) upregulation that narrows until it is along the midline and then further constricts to form a single spot, consistent with experimental observations (Fig. 3A-C,F) (Reed et al., 2007). The eyespot location formed near the observed experimental position using boundary conditions consistent with *in situ* observations (Fig. 3A-D). We discuss the specific choice of boundary conditions in the Materials and Methods. During the whole dynamics, A_1_ and A_2_ were spatially anti-correlated, in agreement with observations of Arm and *dpp* anti-colocalization.

A crucial observation from this model was that the position, size and shape of the spot were sensitive to Dll activity (parameter *K*) and A_2_ production rate (parameter α) with eyespot centers emerging at high *K* and α (Figs S8, S9A). At lower values of *K* and α, the reaction between activator and substrate was not sufficiently strong to overcome degradation of the activator and no eyespot centers formed.

### The Gray–Scott model accurately replicates eyespot formation in mutant clones

**Fig. 4.**
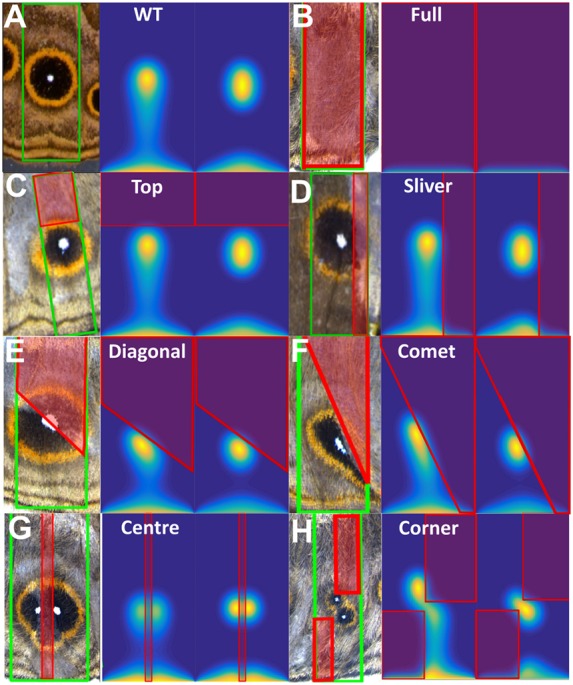
**Reaction-diffusion simulations of wing sectors where part of the sector has no ‘activator’ function.** (A-H) For each panel, left image shows the experimental data. The right shows the *in silico* results after 72 h and 144 h, orientation of compartment and parameters are the same as in Fig. 3A. The region inside the red boxes in each image (except in A), represent the *Dll* mutant region, where *K=0*. The region contoured in green corresponds to the wing cell contours. See text for description of each phenotype. See Fig. S12 to see how red and green contours are defined. WT, wild type. See Movies 1 and 2 for examples of detailed dynamics for A and G.

We modeled *Dll* exon 3 mutant clones as domains in which *Dll* cannot be activated by A_1_. Patches of *Dll* mutant cells were created within a simulated wing sector field by setting *K* to zero (Fig. 3G) (compare wild-type wings in Fig. 4A with red outlined regions in Fig. 4B-H; Fig. S12). Outside this mutant patch, the reaction parameters and boundary conditions remained unchanged. We assumed that A_1_ can diffuse within the *Dll* null region and that diffusion and production of A_2_ are not affected in that same region. We modeled seven *Dll* mutant clones in which the mutant cells are present in different parts of the wing sector: (1) ‘Full’, covering the whole sector; (2) ‘Top’, covering the upper region of the wing compartment; (3) ‘Sliver’, present along one of the side veins; (4) ‘Diagonal’, distal from the wing margin; (5) ‘Comet’, distal from the wing margin but including part of the margin; (6) ‘Center’, present along a stripe at the center; and (7) ‘Corner’; present in two opposite corners of the sector (Fig. 4B-H; Fig. S13). Our model was able to closely reproduce all the crispant phenotypes. For each phenotype, we had the correct number of eyespot centers differentiated and they were positioned in close accordance with our experimental observations. For comparison, we performed simulations of the same clones using the Gierer–Meinhardt activator-inhibitor type model, which reproduced qualitatively most of the observed *Dll* crispant phenotypes, but did not have anti-colocalization of the two morphogens (A_1_ and A_2_) (Figs S13-S16, Table S7, supplementary Materials and Methods).

**Fig. 5.**
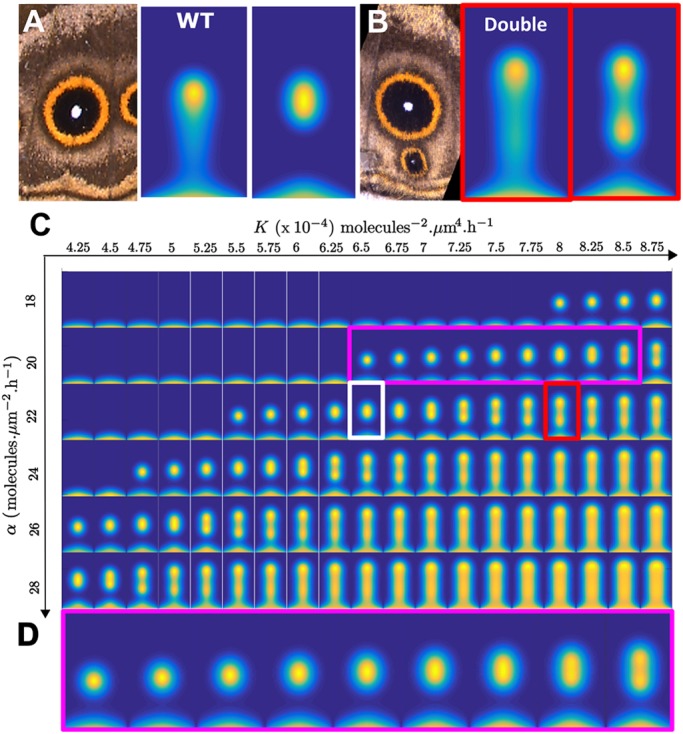
**Perturbations of the Gray-Scott model and the *K*-α phase diagram reveal high sensitivity to changes in *Dll* functionality.** (A) Wild-type (WT) spot at t=72 h and t=144 h. The parameters used correspond to the white rectangle in C. (B) Increasing *K* leads to the appearance of a second spot. The parameters used correspond to the orange rectangle in C. (C) Phase diagram of A_1_ at t=144 h for different *K* and α parameters. (D) Enlargement of the pink boxed area in C. Spot size increases when *K* increases with other parameters fixed, until spot splitting at high *K*.

Alternative splicing of exon 2 is associated with the differentiation of two eyespots and with comet-shaped eyespots. These phenotypes do not show associated pigmentation defects, and, thus, it is unclear the extent or region of the *Dll* mutant clone that produced them. Therefore, we modeled these mutants by assuming that cells expressed a functional truncated Dll protein across the whole wing sector, which degraded more slowly than its wild-type version, effectively resulting in increased *K* (supplementary Materials and Methods; Fig. 5A,B). Keeping all other parameters fixed, increasing *K* led to a spot size increase, until a threshold value *K*_c_ is reached. Above this threshold, the spot splits vertically into two smaller spots (Fig. S14). This phenotype is very similar to the phenotypes observed in Fig. 2F,I. Further increasing *K* resulted in the double-spot phenotype turning into an extended finger pattern, close to the observed comet phenotype (Fig. 2G; Figs S9, S10).

Experimentally, it is known that reducing *Dll* expression across the whole wing results in reduced eyespot size (Monteiro et al., 2013). Keeping our model parameters fixed, we re-ran the simulations with reduced values of *K*, which corresponds to reduced Dll production (Fig. 5C,D). The simulations support this experimental finding by showing that reducing *K* also results in smaller eyespot centers that would lead to smaller eyespots (Fig. 5D). Our model is able to replicate both wild-type behavior and observed phenotypes in a range of crispants. Importantly, this can be achieved through the tuning of a single parameter *K*, which effectively describes *Dll* activity, i.e. our model fitting does not require extensive parameter variability. Therefore, we are confident that we are not over-fitting the model to the data.

## DISCUSSION

Gene expression studies have shown a positive correlation between *Dll* expression and the number and size of eyespots that differentiate on the wings of different butterfly species, including *B. anynana* and *V. cardui* (Oliver et al., 2012, 2014; Saenko et al., 2011; Zhang and Reed, 2016)*.* During the larval stages, *Dll* is expressed in the center of the wing sectors where eyespots will develop, and is absent from the wing sectors where eyespots will not develop (Monteiro et al., 2013; Zhang and Reed, 2016). In a recent study, Zhang and Reed (2016) found that CRISPR-Cas9 targeting of *Dll* exon 2 in *V. cardui* led to ectopic eyespots in wing sectors that normally display no eyespots, leading to the proposal that *Dll* must be a repressor of eyespot development. Mechanistically, however, this result is difficult to explain, as pointed out by the authors. Why would an eyespot repressor gene be naturally absent in sectors without eyespots and present in sectors with eyespots?

To explore this conundrum, we replicated these experiments in *B. anynana.* Similar to Zhang and Reed, we found that targeting the same regions of exon 2 resulted in butterflies with ectopic eyespots in addition to butterflies with missing eyespots, the latter of which was not observed in *V. cardui*. By exploring the effect of the guide RNAs on cDNA sequences obtained a few days after embryonic injections, we found that disruptions in exon 2 produced transcripts completely lacking this exon, regardless of whether disruptions occurred in the 5′UTR or coding region of this exon. In contrast, several indels, but no exon skipping, occurred when we targeted exon 3, indicating that these disruptions led to a non-functional product. Furthermore, we only observed ectopic eyespots when targeting exon 2, suggesting that development of ectopic eyespots was a consequence of this exon-splicing event.

A number of recent studies have shown that CRISPR-induced mutations can lead to alternative splicing and even gain-of-function phenotypes (Kapahnke et al., 2016; Lalonde et al., 2017; Mou et al., 2017). A recent study by Rajaratnam et al. found remarkably similar results to our own, but in sepsid flies (Rajaratnam et al., 2018). They showed that CRISPR targeting of *Dll* exon 3 produced flies with missing sternite brushes, yet targeting exon 2 produced both missing and ectopic brushes. Furthermore, they also observed exon skipping when targeting exon 2 and found an association between gain of an ectopic abdominal sternite brush and mutations within an ESE. As the top predicted ESEs for exon 2 landed within the regions where we designed our guide RNA sequences it is possible that disruptions to this ESE could explain the aberrant splicing we observed.

Our results suggest that CRISPR-Cas9 targeting of exon 2 led to a truncated but potentially functional *Dll* transcript utilizing one of the start codons present in exon 3 to produce an open reading frame with an intact homeodomain (Fig. S6). Rajaratnam et al. (2018) demonstrated using an *in vivo* protein synthesis assay that a *Dll* transcript missing exon 2 but with a functional homeodomain can be translated from an alternative initiation codon to produce a protein of the predicted size. In both studies, however, it still remains unclear how a truncated Dll protein could be associated with ectopic structures.

**Fig. 6.**
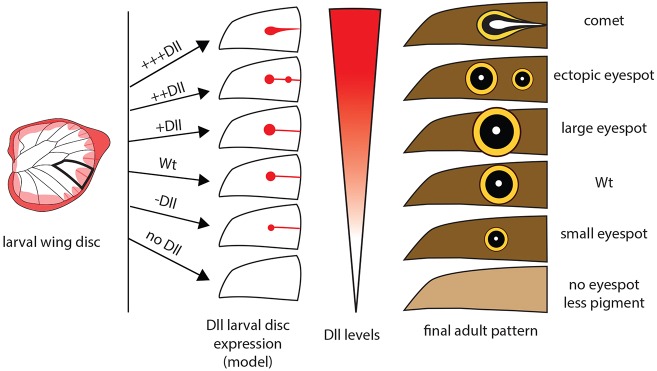
**Schematic of proposed eyespot phenotypes along a gradient of Dll expression in the larval wing disc.** When Dll is absent or at low levels, no eyespots are formed. As Dll expression increases, eyespots are formed and eyespot size correlates positively with Dll levels. Above a certain threshold of Dll expression, the Dll finger (along the midline) splits forming multiple eyespots. At higher expression of Dll, a persistent Dll finger is formed and the comet phenotype is produced. Wt, wild type.

Compared with model organisms such as *D. melanogaster*, for which numerous tools for temporal and spatial regulation of gene function have been developed in homogeneous genetic (transgenic) backgrounds, here we relied instead on random spatial perturbations of Dll function (of variable genetic basis and penetrance) to study a complex morphogenetic wing patterning process. Although this approach has limitations, the spatial complexity and diversity of the color patterning that was obtained with the crispant individuals provided a meaningful test for our theoretical model. Using this model, we gained multiple insights into the role of Dll in the process of eyespot initiation and the conditions under which ectopic eyespots may arise. In particular, we could test the effects of changes in Dll expression on eyespot formation, which are not easily accessible experimentally (Fig. 6). The splitting of eyespot centers and the anti-localization of Arm and *dpp* expression suggests that activator-substrate models, such as the Gray–Scott model, or grass-fire model (Nijhout, 2017), may be more applicable to modeling the establishment of eyespot centers than Gierer–Meinhardt activator-repressor models used previously.

Our simulations using the Gray–Scott model predict that eyespot duplications could occur if the rate of Dll degradation is reduced across the wing cell, essentially leading to Dll overexpression. This led us to speculate that expression levels of *Dll* would be higher in embryos injected with Sg1 (exon2) relative to Sg3 (exon3). Our findings, however, did not support this hypothesis, possibly owing to the overall low expression levels of *Dll* during embryonic development. Alternatively, it is possible that splicing does not impact gene expression levels but instead alters downstream processes affecting translation efficiency (Nishimura et al., 1998). The loss of exon 2 would have resulted in a shorter 5′UTR region, an alteration that is known to increase translation activity owing to removal of inhibitory secondary structures and translational repressive elements (Cavatorta et al., 2011; Wang et al., 2016).

In addition to ectopic eyespots, we also observed comet-shaped eyespot centers, which are associated with strong expression of Dll protein in the spontaneous comet mutant, suggesting an overexpression phenotype (Fig. S10). Our modeling work also predicts comet phenotypes due to the emergence of a stable Dll finger as a result of increased protein expression. Future experiments will need to be performed for a fuller understanding of this phenomenon. In the meantime, it is interesting to note that although only a small number of individuals (14 out of 126 adults) displayed either ectopic or comet eyespots, our results demonstrate that a *Dll* overexpression phenotype can be achieved via disruptions to exon 2 of *Dll*.

A curious observation was butterflies with both missing and ectopic eyespots in different wing sectors on the same wing. By isolating wing tissue from both these regions, we hoped to correlate the frequency of a particular mutation with each specific phenotype using next-generation sequencing; however, similar mutations were associated with both phenotypes. Of course, it is possible that larger deletions extended beyond our primer sites and thus were not captured for amplicon sequencing. It has recently been shown that use of a sgRNA can produce large deletions and complex rearrangements (Kosicki et al., 2018).

Based on our findings, we propose that different phenotypes observed in adult wings may be related to the spatial distribution of each mutant cell clone in the wing sector and from particular mutation events inducing exon skipping, which cannot easily be inferred from the adult wing tissue. Our attempt at genotyping clonal regions revealed the challenges of identifying causal mutations producing crispant phenotypes. One issue with this approach concerns the potential problem of heterogeneous cell populations. As noted by Livraghi et al. (2017), genotyping wing tissue may inadvertently capture cells not even involved in scale differentiation, such as interstitial epithelial cells, neurons and circulating hemocytes. Confidently associating mutations responsible for crispant phenotypes will likely require germline transformation of the causal mutation.

In contrast to exon 2, guide RNAs targeting exon 3 led to missense mutations and missing eyespots, indicating that *Dll* is required for eyespot differentiation. A previous study performed in *B. anynana* had already functionally implicated *Dll* as a positive regulator of eyespot development, but the results were less stark than those reported here. *Dll* downregulated by transgenic RNAi led to smaller eyespots, rather than missing eyespots, whereas its upregulation led to two smaller eyespots appearing on the forewing (Monteiro et al., 2013). *Dll* downregulation failed to remove eyespots presumably because it was implemented during a limited period during late larval development and because it likely failed to eliminate *Dll* transcripts altogether (compared with CRISPR, which can induce complete *Dll*-null clones). The two studies, however, by obtaining essentially the same results via the use of two different approaches, confirm that *Dll* is a positive regulator of eyespot development in *B. anynana* and likely also in other species.

In addition to eyespot center differentiation, we confirmed that *Dll* has additional roles in patterning the parafocal margin elements but the central symmetry system was unaffected. These findings are consistent with known expression patterns of Dll along the wing margin. Our findings also provide further support for the role of Dll in wing melanization, previously shown in *B. anynana* (Monteiro et al., 2013), as well as in *Drosophila biarmipes* and *Junonia orithya* (Arnoult et al., 2013; Dhungel et al., 2016). In *B. anynana*, ectopic expression of *Dll* during the early pupal wing led to patches of darker scales on the wing, whereas *Dll* RNAi led to no observable change in color (Monteiro et al., 2013). The current *Dll* exon 3 crispants, with light-colored patches of pigmentation, lend additional support for this function of *Dll*. This function is also supported by overall Dll expression (albeit at low levels) across all scale-building cells of the pupal wing of *B. anynana* (Fig. 1F).

*Dll* appears to have a further role in scale cell development. In several *Dll* crispants, a specific type of scale, the cover scales, or both cover and ground scales, were missing from patches on the wing. Patches of scales with reduced pigmentation may have been due to weaker *Dll* mutant alleles, whereas those with scales missing may have been due to stronger alleles. This suggests that *Dll* is required for scale development. Scale cells, owing to their pattern of division, differentiation and growth, and expression of an *achaete-scute* homolog, have been proposed to be homologous to *Drosophila* sensory bristles, which share similar characteristics but are restricted to the anterior margin in the fly wing (Galant et al., 1998). In *Drosophila*, *Dll* mutant clones along the wing margin lead to loss of *achaete-scute* expression and loss of bristles (Campbell and Tomlinson, 1998). Our results further strengthen the hypothesis that butterfly wing scales are novel traits that originated from modified sensory bristles, which populated the entire wing blade.

Thus, building on previous functional work in *B. anynana* and findings from the current study, we have developed a schematic model that can explain a diversity of eyespot phenotypes. We propose that variation in levels of Dll expression in the larval wing disc generates a variety of eyespot phenotypes in the adult (Fig. 6). Our model suggests that development of wild-type eyespots requires Dll to be expressed above a certain threshold in the midline of each wing sector to induce eyespot center differentiation. Expression variation above this threshold first regulates eyespot size, and later results in the midline finger splitting into multiple focal points producing ectopic eyespots. Finally, further sustained or increased Dll in the midline leads to formation of the comet phenotype, as seen in species of the genus *Euptychoides* (e.g. *E. albofasciata*). Our model also suggests that the origin of eyespots, often described as a qualitative, saltational process of network recruitment (Monteiro, 2015; Oliver et al., 2012), might have resulted from a simple tuning of Dll expression levels, or of other essential components of the likely underlying reaction-diffusion network, that crossed threshold conditions for eyespot center differentiation.

## Conclusions

Here, we show that CRISPR targeting of Dll can lead to either gain or loss of eyespots, demonstrating a specific role of *Dll* in eyespot center differentiation. Additionally, we show that Dll is also involved in the regulation of melanin pigmentation across the whole wing, not just in the black regions of the eyespot, where its expression is stronger. Our work also suggests a new role of Dll in scale development, and confirms the established role of *Dll* in ventral appendage development. The discovery that CRISPR-Cas9-induced mutations in *Dll* can produce both knockout and gain-of-function phenotypes opens up avenues for further investigation. Future work using transgenic butterflies could explore whether alternative splicing of *Dll* can lead to the loss or gain of butterfly eyespots. Finally, we provide a detailed reaction-diffusion model that accurately describes the dynamics of wild-type and mutant eyespot formation. This model identifies *Dll* as having a crucial role in eyespot formation, in which tuning expression levels of Dll may in part explain variation in butterfly eyespot diversity and, potentially, eyespot origins.

## MATERIALS AND METHODS

### Animal husbandry

*Bicyclus anynana* were reared at 27°C and 60% humidity inside a climate room with 12:12 h light:dark cycle. All larvae were fed young corn leaves until pupation. Emerged butterflies were frozen and then the wings were cut from the body prior to imaging using a Leica DMS1000 digital microscope. Images of wild-type *B. anynana* showing the dorsal and ventral patterns can be found in the Dryad Digital Repository (https://doi.org/10.5061/dryad.2108pm6; Connahs et al., 2019) along with images of all crispants and an Excel file enumerating all crispant wing phenotypes.

### Guide RNA design

Guide RNAs corresponding to GGN_20_NGG (Dll) were designed using CRISPR Direct (Naito et al., 2015). We separately targeted three sites in Dll with two guides targeting exon 2 (in the 5′UTR and coding sequence) and a third guide targeting the homeobox of exon 3 (Fig. 1A). The guide RNAs were created by amplifying overlapping primers (Bassett et al., 2013) (Table S4) using Q5 polymerase (New England Biolabs). One primer contains the T7 promoter sequence and gene target region and the other is a common reverse primer composed of the guide RNA backbone. Constructs were transcribed using T7 polymerase and (10X) transcription buffer (New England Biolabs), RNAse inhibitor (Ribolock), NTPs (10 mM) and 300 ng of the guide template. Final sample volume was 20 μl. Samples were incubated for 16 h at 37°C and then subject to DNase treatment at 37°C for 1 h. Samples were purified by ethanol precipitation and RNA size and integrity was confirmed by gel electrophoresis.

### *In vitro* cleavage assay

The guide RNAs were tested using an *in vitro* cleavage assay. Wild-type genomic DNA was amplified using primers designed to the region flanking the guide RNA target sites. Guide RNA (160 ng), Cas9 protein (322 ng) (stored in a buffer containing 300 mM NaCl, 10 mM Tris-HCl, 0.1 mM EDTA, 1 mM DTT, 50% glycerol pH 7.4 at 25°C) and 10X buffer (1 μl) were brought to a final volume of 10 μl with nuclease-free water and incubated for 15 min at 37°C. The purified amplicon (100 ng) was added and the reaction incubated for a further 1-2 h at 37°C. The entire reaction volume was analyzed on a 2% agarose gel. Cas9 protein was purchased from two suppliers: NEB EnGen Cas9 NLS (exon 2 injections) and PNA Bio (exon 3 injections).

### Embryo injections

Wild-type *B. anynana* adults were allowed to lay eggs on corn plants. Eggs were picked within 1 h of oviposition and immobilized with 1 mm wide strips of double-sided tape in plastic 90 mm Petri dishes. Cas9 protein and guide RNA were prepared in a 10 µl volume and incubated for 15 min at 37°C prior to injection along with 0.5 µl food dye to aid embryo injections (Table 1). The injection mixture was prepared fresh each time from aliquots of Cas9 and kept on ice after incubation prior to injection. The mixture was injected into eggs by nitrogen-driven injections through glass capillary needles. Injected eggs were stored in closed Petri dishes, accompanied by daily re-dampened cotton balls to maintain humidity. After hatching, larvae were reared in small containers for 1 week then moved to corn plants to complete their development.

### Screening and genotyping crispants

Upon emergence, butterflies were immediately stored at −80°C in individual containers. All individuals were screened under a microscope and examined for asymmetric crispant phenotypes. For selected crispants, genomic DNA was extracted from dissected wing tissue displaying mutant clone regions and modified/ectopic eyespots (E.Z.N.A tissue DNA kit). For next-generation sequencing, amplicons shorter than 500 bp incorporating exon 2 or exon 3 were amplified using barcoded primers by PCR (Table S4). The samples were visualized on a gel to confirm the presence of a single band then purified using a Thermo Scientific PCR purification kit. The purified products were quantified using Qubit and sequenced using Illumina Miseq (300 bp paired-end). Exon 3 crispants were sequenced by AITbiotech (Singapore), and exon 2 crispants were sent to the Genome Institute of Singapore. Sequencing coverage was 10,000×. Demultiplexing was performed using an in-house python script (Meier et al., 2015). The fastq files were checked for quality and trimmed using PRINSEQ (Schmieder and Edwards, 2011). The trimmed files were processed using the command line version of CRISPResso (Pinello et al., 2016).

### Detection of alternative splicing and quantitative PCR

RNA was isolated from injected eggs [guides targeting 5′UTR of exon 2 (Sg1) and the homebox domain of exon 3 (Sg3)] and control eggs (no injection) using the Qiagen RNeasy mini kit incorporating a DNase I treatment (Thermo Fisher Scientific). RNA was isolated 2 days after egg injection. For each treatment group, we prepared four replicates of 50 pooled eggs on the same day. To control for developmental timing, we alternated injecting 50 eggs between the two groups (Sg1 and Sg3) for a total of 200 eggs/group. Eggs were placed in a Petri dish of PBS and injected within 90 min of oviposition. After 2 days, eggs were carefully removed from the PBS and briefly transferred to RNAlater (Qiagen) prior to RNA isolation. For each of the 12 RNA samples, 2 µg of RNA was used as input for cDNA synthesis (Thermo Scientific Revertaid First Strand). cDNA was also obtained from 50 pooled eggs injected with Sg2 in a separate experiment using the same protocol. PCR was performed on the cDNA using *Dll* primers spanning exons 1-6 (wild type=1.5 kb product) and visualized on a 1.5% agarose gel. The spliced transcript produced from the guide targeting the 5′UTR of exon was cloned into a pGEM t-easy vector followed by colony PCR using M13 primers to identify colonies carrying this product. The short insert (∼1 kb) was amplified using the Big Dye sequencing kit (Thermo Fisher Scientific) and sequenced. To confirm reproducibility of exon skipping for both Sg1 and Sg2, we repeated the embryo injections for Sg2 to obtain more replicates (*n*=4, 50 pooled embryos per replicate). Following cDNA synthesis and PCR as described above the products for all cDNA samples for each guide were run on a 1% agarose gel.

qPCR was performed on the cDNA from embryos injected with Sg1 or Sg3 (representing exon 2 and exon 3 disruptions). Primers were designed using Primer3 plus for *Dll* exon 1 and an internal control gene EF1 alpha. Relative expression was performed using a qPCR mastermix (Kapa SYBR Fast Uni) and 4 ng of cDNA from four biological replicates and two technical replicates in a single experiment. Four biological replicates were tested to ensure sufficient statistical power to detect expression differences. The reaction was set up following the manufacturer's instructions and run on a Bio-Rad thermocycler. Relative expression software tool (REST) was used to analyze the expression data (Pfaffl et al., 2002).

### *In-situ* hybridization

*In-situ* hybridization was performed on fifth instar larval wing discs. Wings were dissected in cold PBS and transferred into fixative containing 4% formaldehyde. After proteinase K treatment, peripodial membranes were removed using fine forceps. The wings were then gradually transferred in increasing concentrations of pre-hybridization buffer in phosphate-buffered saline with Tween 20 and incubated in pre-hybridization buffer at 65°C for 1 h before transferring into hybridization buffer containing 70 ng/ml probe. Hybridization was carried out in a rocking-heating incubator at 65°C for 20 h. After hybridization, wings were washed five times in pre-hybridization buffer for 20 min at 65°C. Blocking was carried out using 1% bovine serum albumin in PBST. Anti-digoxygenin AP (Roche; 1:3000) was used to tag digoxygenin-labeled probes. NBT/BCIP (Promega) in alkaline phosphatase buffer was used to generate color. Imaging was carried out using a Leica DMS1000 microscope with LAS v4.9 software.

### Antibody staining

Fifth instar larval wing discs were dissected in cold PBS and incubated in fix buffer [1 M PIPES (pH 6.9) (500 mM), 1 mM EGTA (pH 6.9) (500 mM), 1% Triton X-100 (20%), 2 mM MgSO_4_ (1 M), 4% formaldehyde (added just prior to the addition of the discs) (37%), H_2_O to a volume of 30 ml] for 35 min, washed four times in cold PBS and blocked using block buffer [50 mM Tris (pH 6.8) (1 M), 150 mM NaCl (5 M), 0.5% IGEPAL (NP40) (20%) and 5 mg/ml BSA; brought to a final volume of 40 ml with H_2_O] for 2 days. Wings were stained against Armadillo using an unpublished primary polyclonal antibody [294 rabbit anti-Arm; a gift from Nicholas Tolwinski using the same protocol as previously described (Colosimo and Tolwinski, 2006)] at 1:10 and secondary antibody (Alexa Fluor 488-conjugated goat anti-rabbit, A-11008, Thermo Fisher Scientific) at 1:800. Wings were then mounted on ProLong Gold Antifade Mountant (Thermo Fisher Scientific) and imaged under a Zeiss Axio Imager M2 using Zen 2012 software.

### Modeling details

#### Model network

Although specific details of the interactions between Wg, Dll and Dpp are not known in *B. anynana*, there has been extensive work on these interactions in *Drosophila.* High Wg activity is correlated with *dpp* repression (Johnston and Schubiger, 1996). Wg and Dpp can activate *Dll* in a combinatorial manner (Estella and Mann, 2008). Ectopic Dll expression in the proximal region of ventral appendages induces non-autonomous duplication of legs and antennae by the activation of Wg and Dpp (Gorfinkiel et al., 1997). From our data in the butterfly wing compartment, we see that Dll and Arm are colocalized and Dll and dpp are anti-colocalized. Therefore, the wiring diagram shown in Fig. 3E is consistent with current available evidence.

#### Parameter estimation

We modeled a wing sector bordered by veins and containing a single eyespot as a rectangle with typical width *L_x_*=150 μm and length *L_y_*=262 μm (Fig. 3D), (Sekimura et al., 2015). We used degradation and diffusion rates for both A_1_ and A_2_ close in magnitude to those measured for Wg and Dpp, respectively, in the *Drosophila* wing disc (Kicheva et al., 2007). Owing to the longer time scales involved in eyespot patterning, both degradation and diffusion rates were assumed to be smaller than in *Drosophila* (therefore, we explored values varying by a factor of 0.1-1). In line with experimental observations where we observed a decrease in *dpp* (Fig. S7) at late larval stage, we decreased α by 25% at time t=60 h in the simulation.

We present in Fig. 4 the results of the simulations for the different *Dll* mutant conditions. Results are shown for the parameter set that maximizes the matching between eyespot number and location(s) in the wing compartment between the simulations and the experimental data. The same parameter set was used in all simulation results shown. To model exon 2 mutations, we increased *K*, which corresponds to either increasing *Dll* expression levels or decreasing its degradation rate (see supplementary Materials and Methods).

#### Boundary conditions

Boundary conditions were implemented based on the *in situ* hybridization and immunostaining for *dpp* and Arm (Fig. 3A,B). The wing margin was modeled as a source term of Wnt signaling as Arm is present along the wing margin of *B. anynana* and *wg* is also present along the wing margin of other butterflies (Martin and Reed, 2010). As *dpp* is absent along the wing veins (Fig. 3A), we modeled the veins as sinks for both Wnt and Dpp signaling, which helped to confine the activator and substrate to the central part of the wing sector in a finger-like pattern (Fig. 3D,F). Having a zero flux boundary for Wnt across the wing veins does not significantly alter the simulation results. We require the substrate concentration to deplete to zero at the wing sector margins. Experimentally, we see no *dpp* expression at the wing veins during larval wing development, as shown in Fig. 3A. These conditions differ from those used by Nijhout (1990) and by Sekimura et al. (2015) where the proximal cross-vein and lateral veins are the only sources of activator and inhibitor.

#### Initial conditions

At t=0 h, there are no activator and substrate in the wing sector. At t=0 h, A_1_ starts to diffuse from the wing margin to the wing sector, and the substrate A_2_ is produced by all cells in the wing sector. We assume detailed balance in the reactions, which can lead to spot formation in the Gray–Scott model (supplementary Materials and Methods).

## Supplementary Material

Supplementary information
