# Supplementary material for: Activation of butterfly eyespots by Distal-less is consistent with a reaction-diffusion process
Source: Development. 2019 May 9;146(9):dev169367. doi: 10.1242/dev.169367 (PMC6526720; doi:10.1242/dev.169367)
Supplement: Supplementary information [file develop-146-169367-s1.pdf]

Fig. S1. *In vitro* cleavage assay results on Exon 2 and Exon 3 Dll amplicons (100ng input DNA) showing strong cutting efficiency of Sg1 and Sg3. Very weak bands were observed on the gel for Sg2, however the intensity of the original product is significantly reduced relative to the control indicating that some cleavage has occurred as supported by crispant phenotypes induced by this guide. Control represents 100ng of the Dll amplicon only.

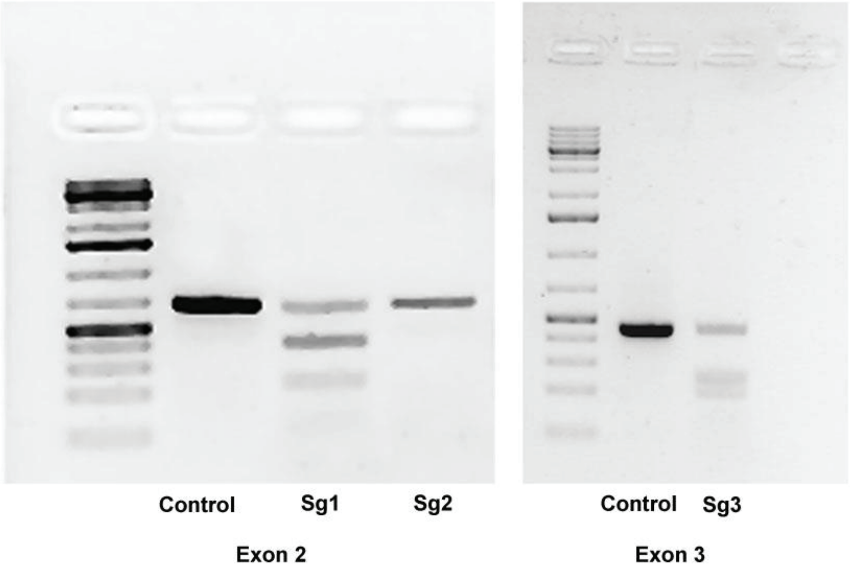

Development • Supplementary information

Fig. S2. Close up image of gray-blue ground scales from crispant C (see Fig. 2) injected with Sg3 targeting exon 3. Magnification 12X.

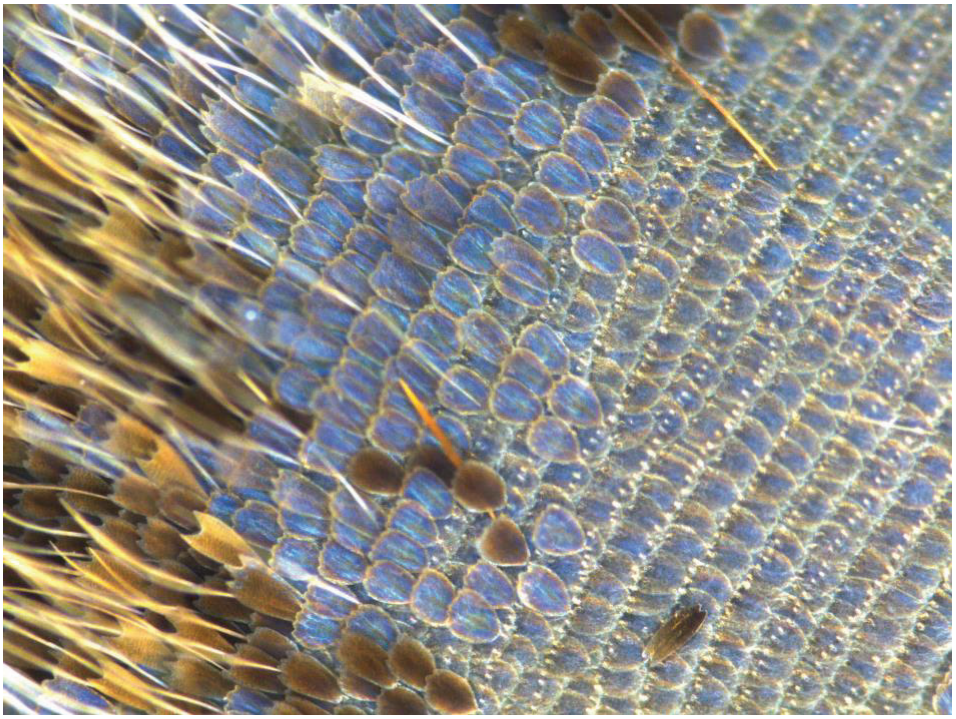

Fig. S3. Appendage defects from exon 3 and exon 2 crispants. (**A**) wild-type legs (left) and exon 3 crispant (right) showing severely deformed and truncated legs. (**B**) Exon 3 crispant with missing tip of antenna. (**C**) Exon 2 crispants showing fused and truncated antenna. (**D**) Exon 2 crispant showing deformed leg with proximal region fused (red arrow). (**E**) Exon 2 crispant with severely deformed and truncated legs.

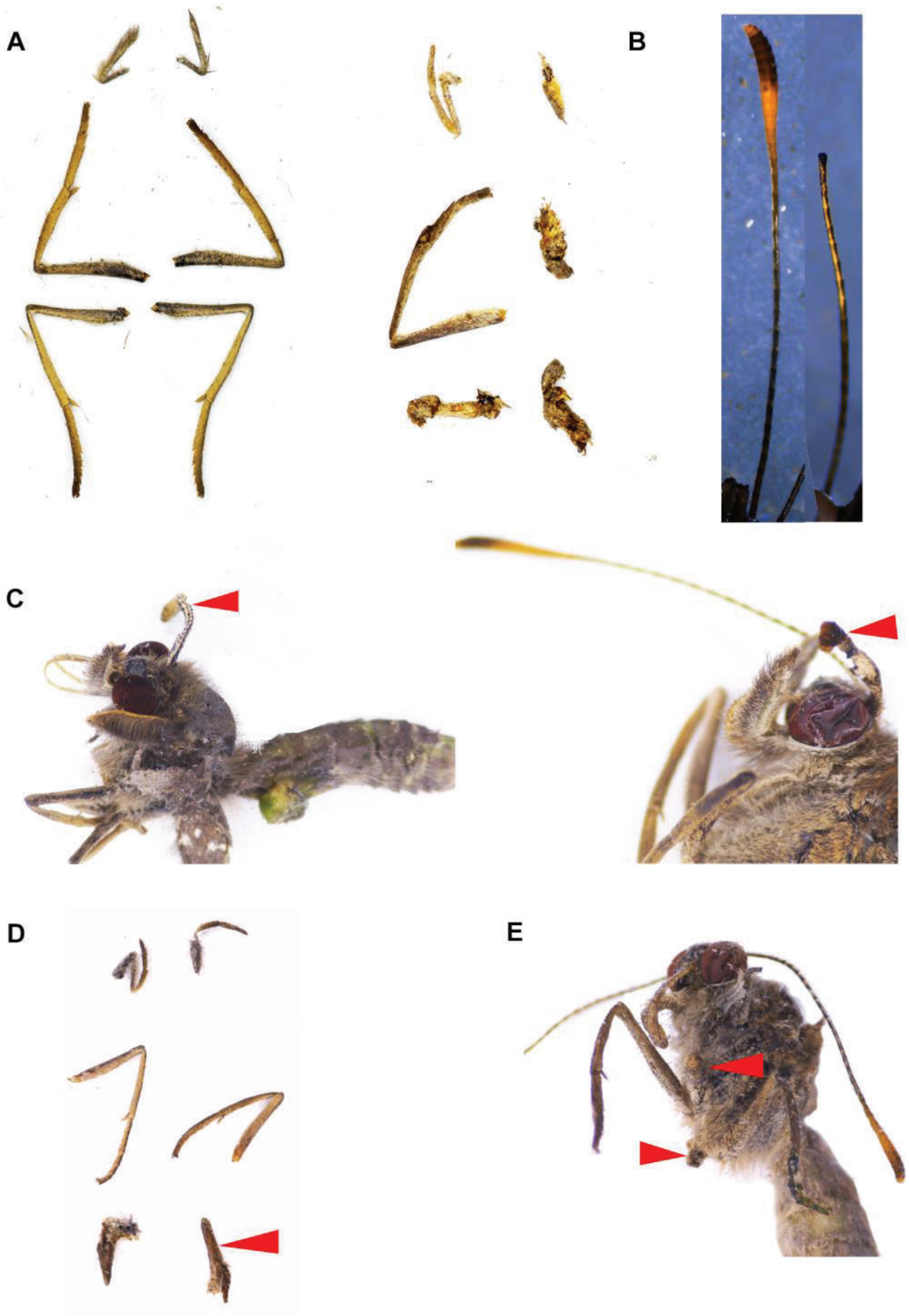

Fig. S4A. CRISPResso analysis of next generation amplicon sequencing results of mutations around the cleavage site for selected Exon 3 crispants (refer to Fig. 2). The figures illustrate the diversity and proportions of mutations induced by the CRISPR-cas9 system within the same tissue. (Orange: SgRNA on reverse strand, Red: PAM). We believe that CRISPResso is overestimating allele diversity in several of the alignments show below (without manual curation).

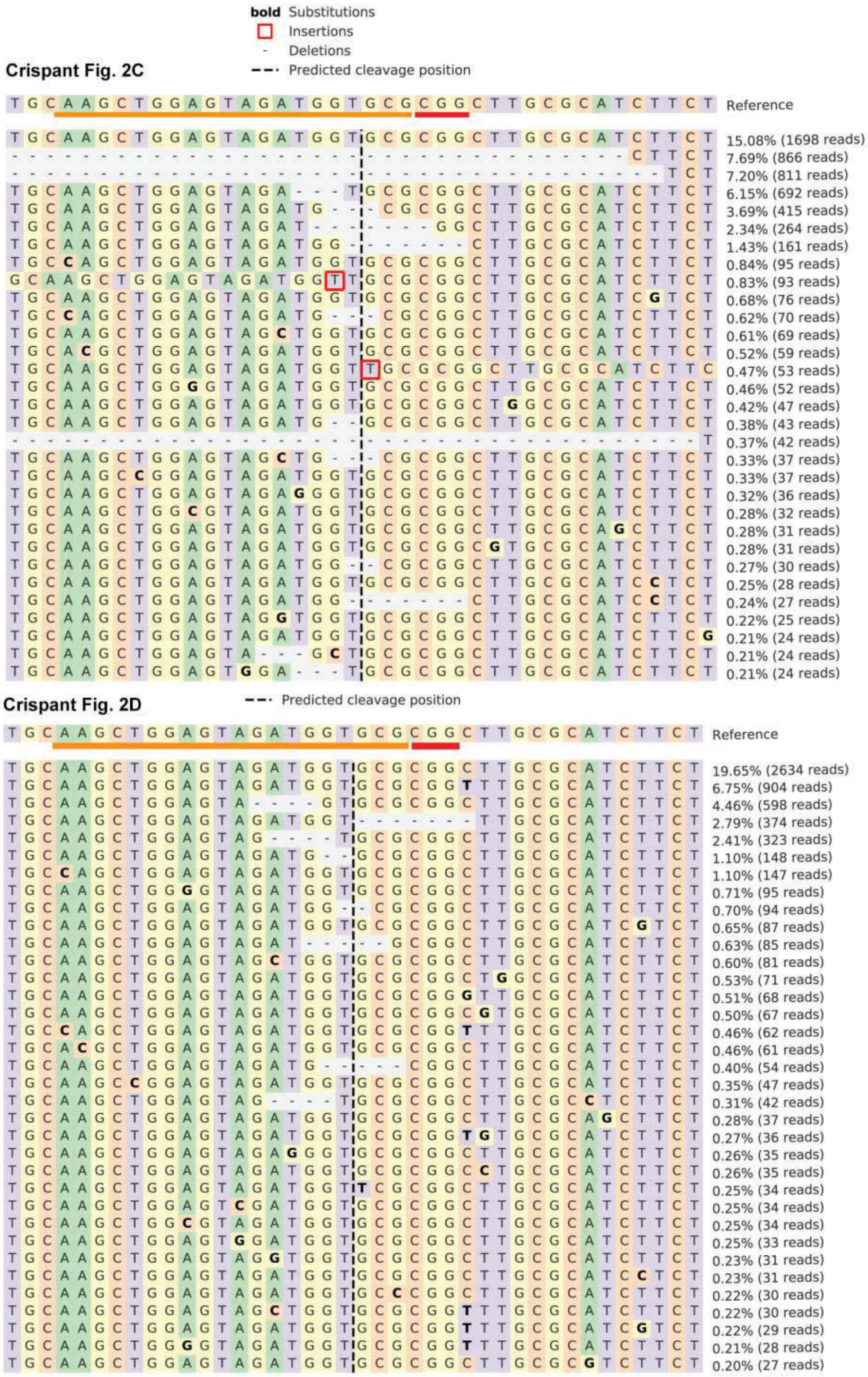

Development • Supplementary information

Fig. S4B. CRISPResso analysis of next generation amplicon sequencing results of mutations around the cleavage site for selected Exon 2 crispants (refer to Fig. 2). The figures illustrate the diversity and proportions of mutations induced by the CRISPR-cas9 system within the same tissue. (Orange: SgRNA on forward strand, Red: PAM).

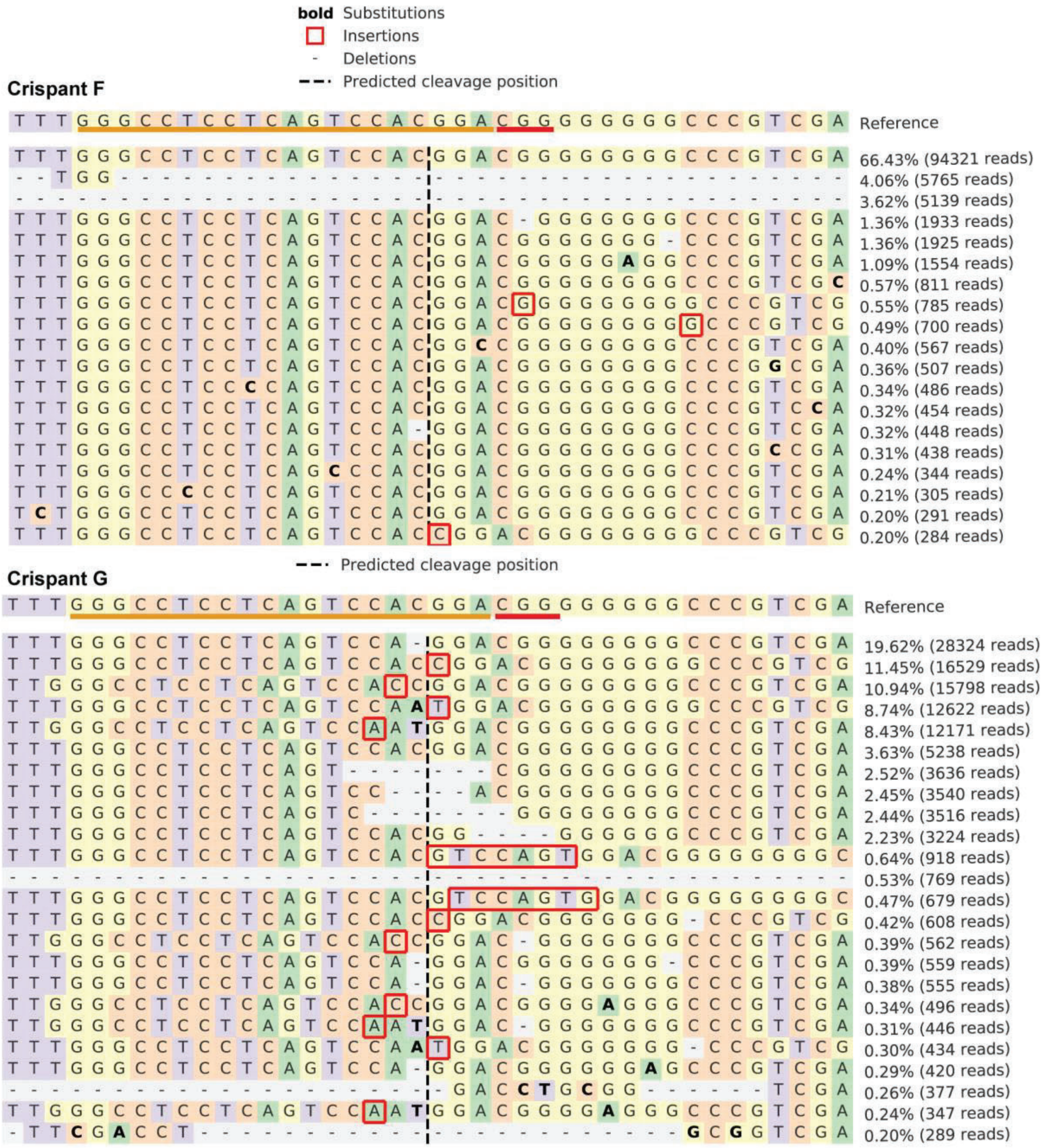

Fig. S4C. CRISPResso analysis of next generation amplicon sequencing results of mutations around the cleavage site for selected crispants (refer to Fig. 2). The figures illustrate the diversity and proportions of mutations induced by the CRISPR-cas9 system within the same tissue. (Orange: SgRNA on forward strand, Red: PAM).

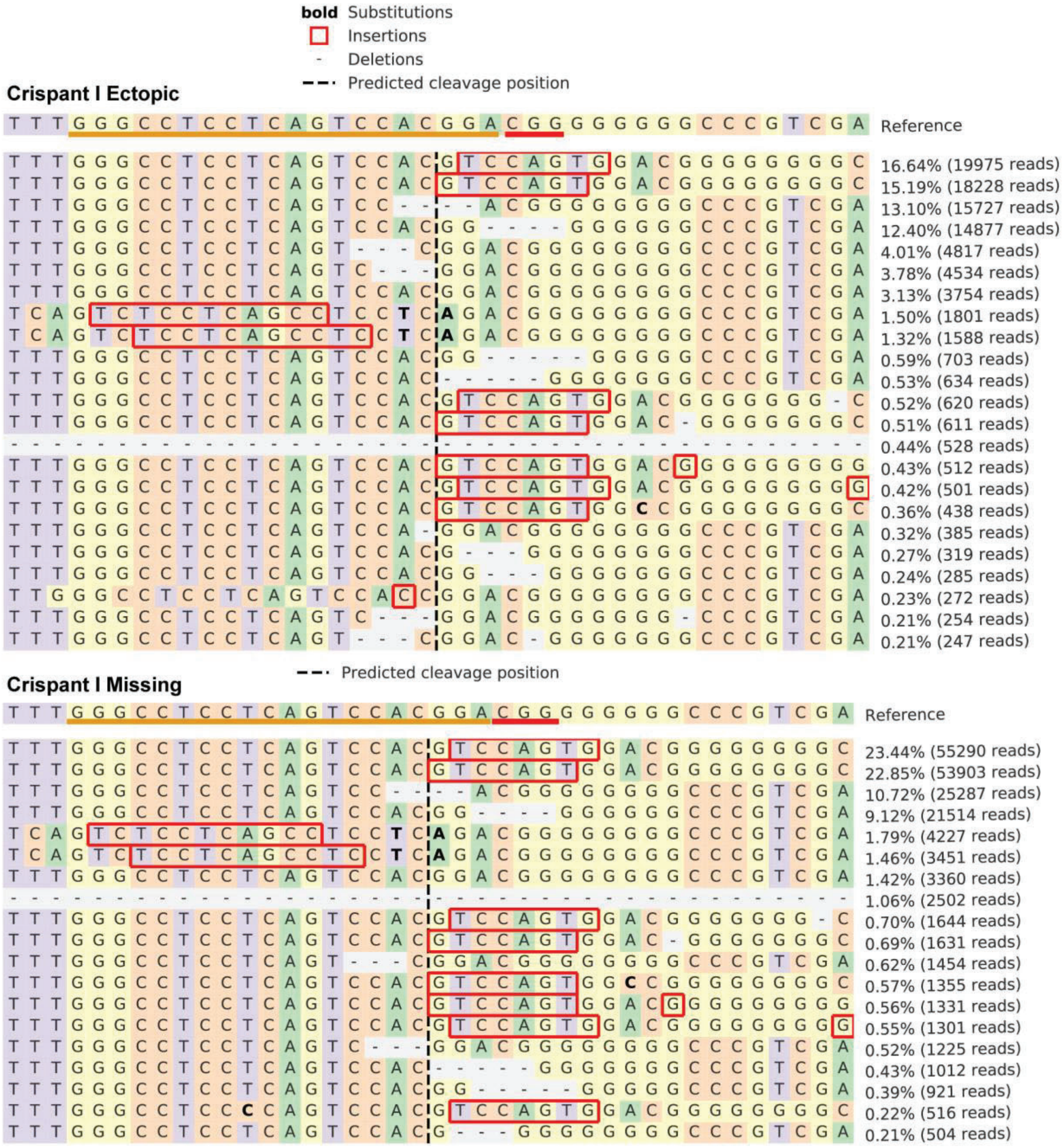

Development • Supplementary information

Fig. S5. Graphical representation of CRISPResso results showing the spatial distribution of each mutation around the cleavage site for selected crispants (see Fig. 2), revealing wide variation in the size of indels even when using the same RNA guide. It is possible that some of the indels called by the software that are located far from the PAM site are in fact SNPs.

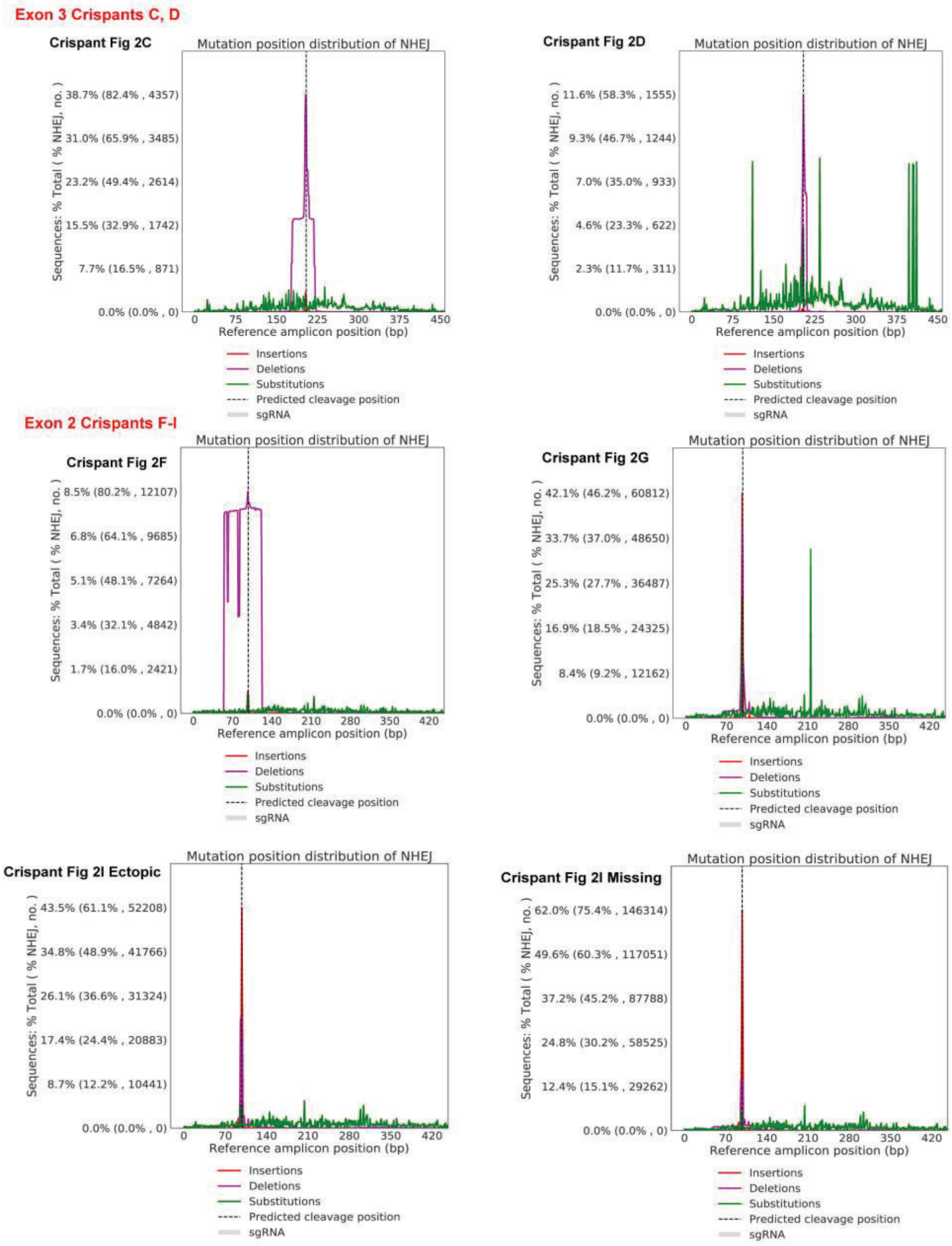

Development • Supplementary information

Fig. S6A. Gel image of PCR using Dll primers spanning exons 1 – 6 on cDNA from a pool of 50 embryos (48hrs) injected 60-90 mins AEL with each guide. First lane shows cDNA from control embryos (no injection). Embryos injected with Sg1 and Sg2 targeting the 5'UTR and coding sequence of exon 2 respectively show a novel product around 1kb in addition to the wild-type product at 1.5kb. Sg1 also shows a slightly fainter band around 1200 bp likely representing another truncated transcript. Embryos injected with Sg3 targeting exon 3 only show the wild-type product. The alignment shows the sequencing results of the cloned 1kb amplicon with a large deletion created by the complete splicing out of exon 2. When translated this product displays the 5'UTR of exon 1 and an open reading frame from exon 3 – exon 6 (amino acids shaded in pink) which potentially could be processed as a functional protein also containing the complete homeodomain (amino acids outlined in red box). The blue-boxed amino acid (K) indicates the start of exon 3. Bottom panel shows the wild-type Distal-less transcript, showing the boundaries between exons 1-2 and 2-3 along with annotations of the homeodomain and putative start codons which produce an open reading frame from exon 3, and potentially a functional protein in the truncated version of Dll.

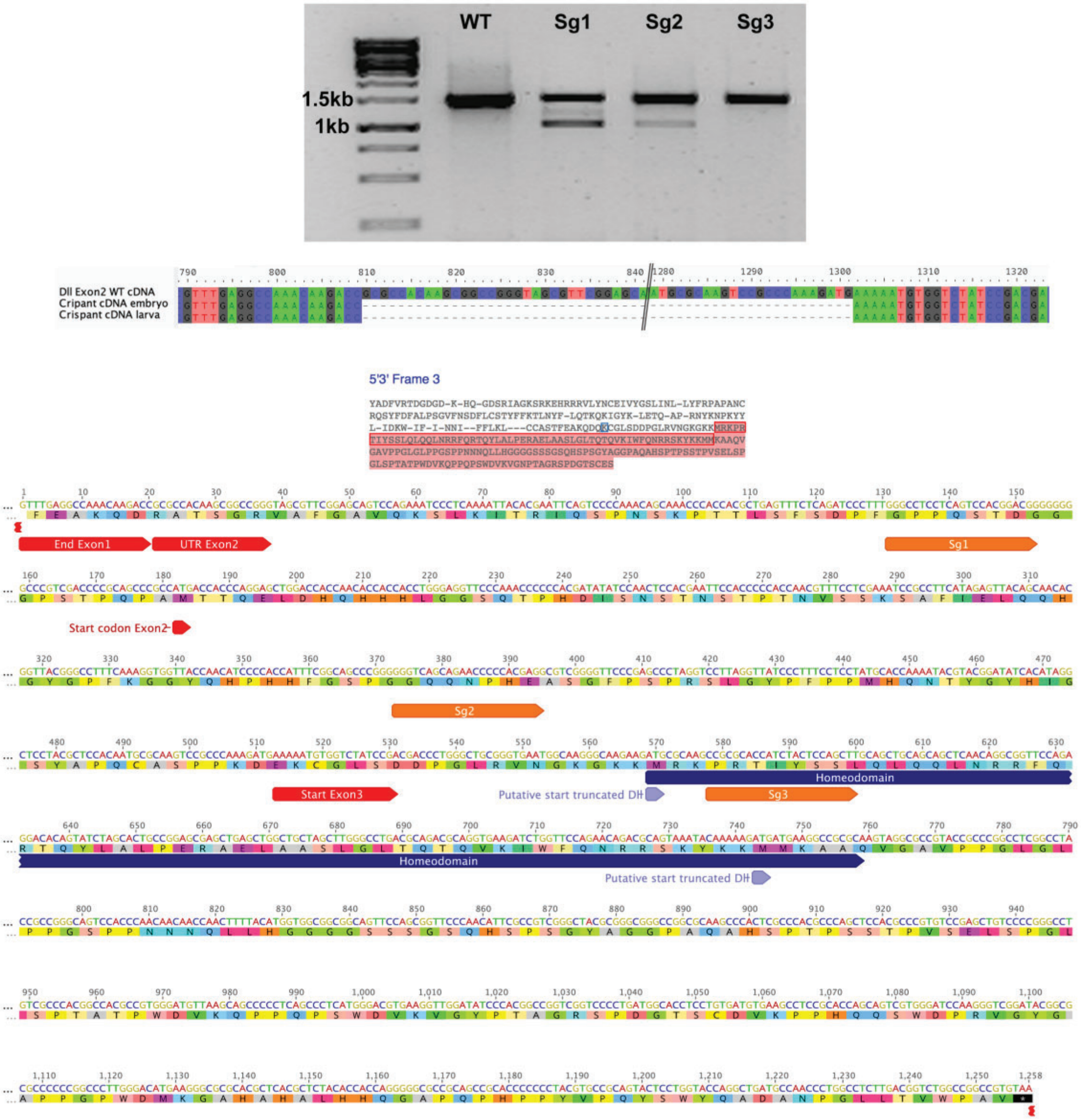

Development • Supplementary information

Fig. S6B. Biological replicates of RT- PCR using Dll primers spanning exons 1 – 6 on cDNA from wild-type embryos and embryos injected 60-90 mins AEL for each guide. Each biological replicate represents a pool of 50 embryos. Results show evidence of additional truncated products only for Sg1 and Sg2.

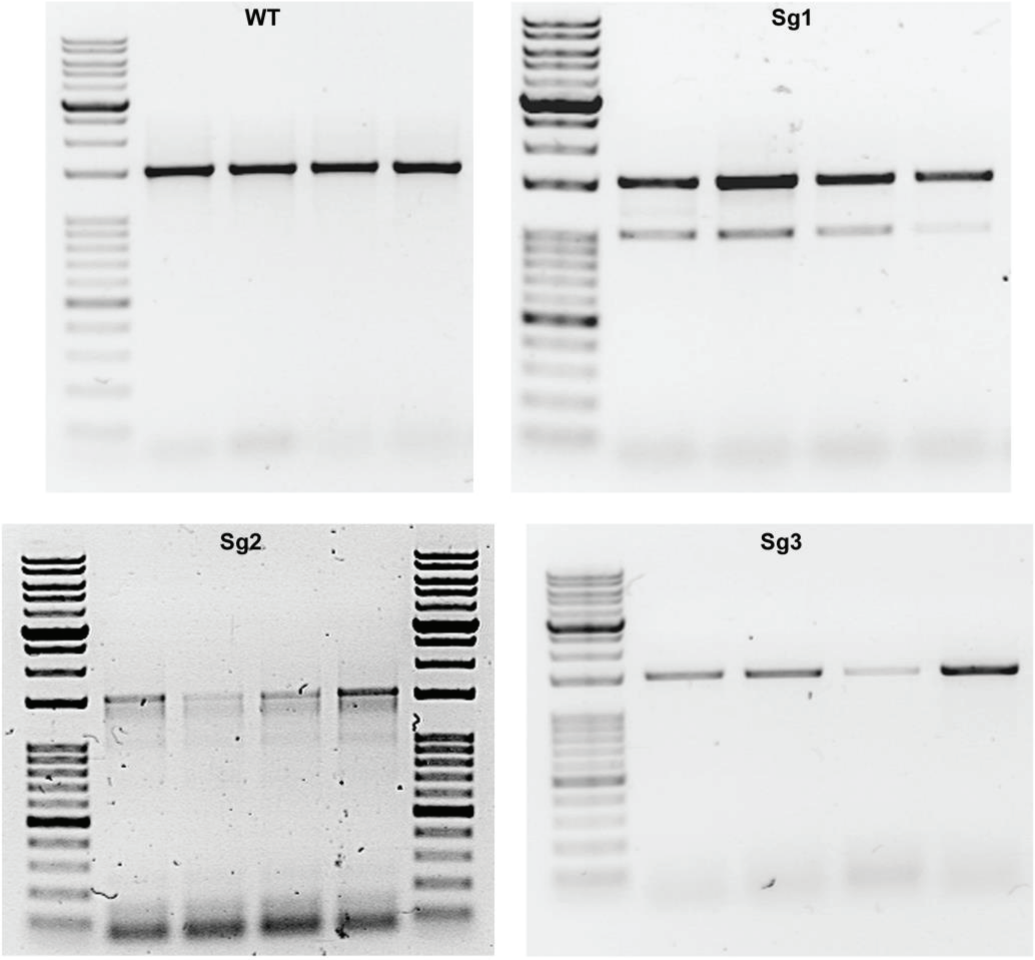

Development • Supplementary information

Fig. S7. *In situ*-hybridization of *dpp* at different larval stages (developmental staging from (Reed et al., 2007)). (**A**) Early stage (stage 0.25), *dpp* is present in a stripe separating anterior and posterior wing compartments consistent with the *dpp* stripe observed in *Drosophila* wings (Akiyama and Gibson, 2015) (n = 4 wings). (**B**) Few tracheae start to reach the border lacuna (stage 1.0)**,** *dpp* is present in wing compartments and starts to deplete from a central finger region and from the spot region (n = 8 wings) (**C**) Majority of tracheae reach border lacuna (stage 2.0), *dpp* is depleted from the spot region (n = 6 wings for C and D) (**D**) Most tracheae extend into border lacuna (stage 2.5), *dpp* is absent from most of wing compartments. (**E**) Arm staining (stage 2.0), Arm is present in a central finger pattern (n = 8 wings) and in spot regions (n = 3 wings). Expression of Armadillo corresponds to expression of *wingless* in the margin and eyespot in *Bicyclus anynana* wings (Özsu et al., 2017). **(F)** pSMAD (signal transducer for dpp) antibody localization (at stage 1.75) mirrors *dpp in situ* staining, and shows anti-colocalization with Arm.

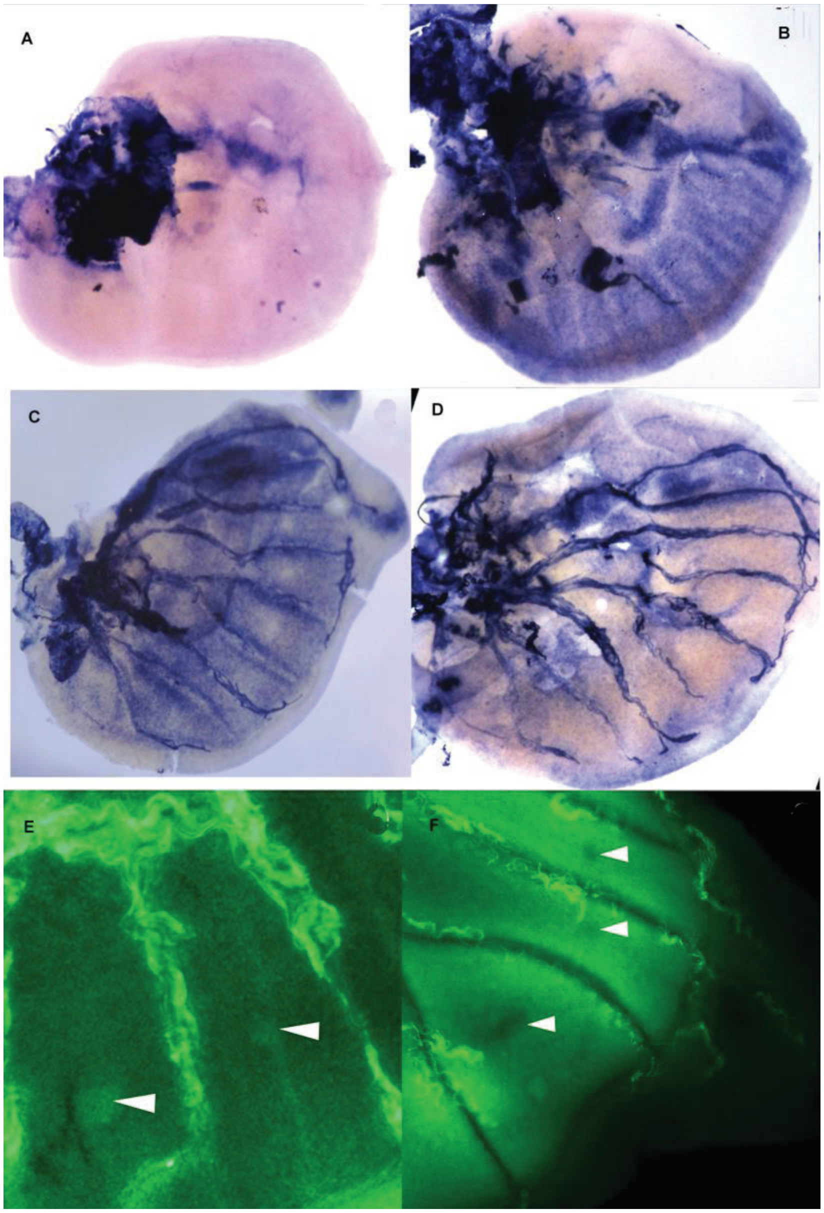

Development • Supplementary information

Fig. S8. Different types of stationary patterns were obtained without reducing α at an intermediate state. (**A**) **Margin band**, the reaction rate is not high enough to trigger activator propagation, A_1 diffuses and degrades from the margin. (**B**) **V-shape** in the margin, reaction starts and converges to a v-shape stationary state. (**C**) **Single spot**, reaction starts in a finger and the finger converges to a stable single spot. (**D**) **Finger-spot**, reaction starts in a finger and the finger converges to a finger-spot state. (**E**) **Finger**, the reaction forms a finger which invades all the wing cell height. (**F**) **Large finger**, the reaction forms a finger which invades all the wing cell height and width. (**G**) **Double spot**, reaction starts in a finger and the finger converges to a stable double spot state.

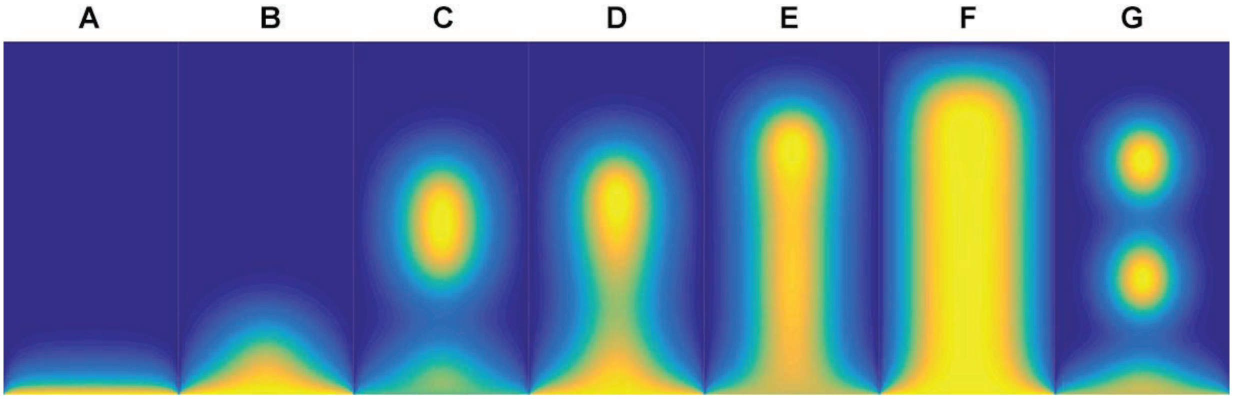

Development • Supplementary information

Fig. S9. Phase diagram of $[A_1]$ field with varying α and K after t=6 days of simulation time with $D_1 = 0.01 \mu m^2/s$, $D_2 = 0.12 \mu m^2/s$, $k_1 = 0.1 \times 10^{-3} s^{-1}$, $k_2 = 0.08 \times 10^{-3} s^{-1}$. (**A**) Phase diagram showing the transition from no spot pattern to spot and finger patterns. (**B**) Increasing *K* with all other parameters fixed leads to a continuous transformation from a single spot to a double spot and then to a finger pattern (magenta region in **A**).

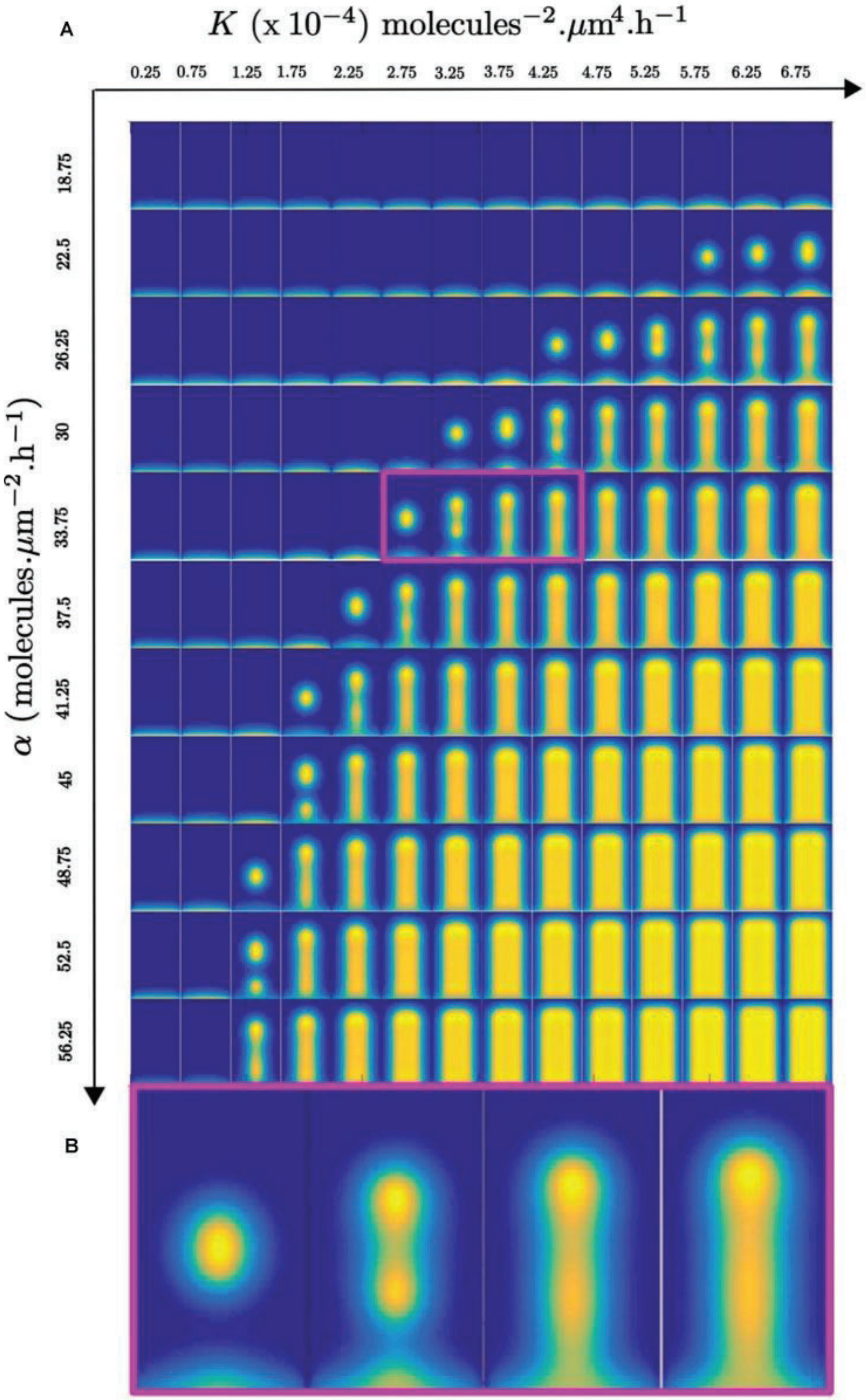

Development • Supplementary information

Fig. S10. Spontaneous lab mutants (**A,B**) showing comet eyespot phenotype with a teardrop drop shape. Interestingly, some wing sectors also show duplicated eyespots. (**C,D**) Examples of Dll antibody staining on larval wings for spontaneous lab comet mutants. Images illustrate the expanded expression domain of Dll in eyespot centers and suggest that Dll overexpression may contribute to this phenotype. (**E**) Wild-type hindwing. (**F**) Dll antibody staining of mid 5th instar wild-type wings showing thin stripes of Dll fingers terminating in a spot. Methods for antibody staining of these wings (previously unpublished) are described in (Monteiro et al., 2003).

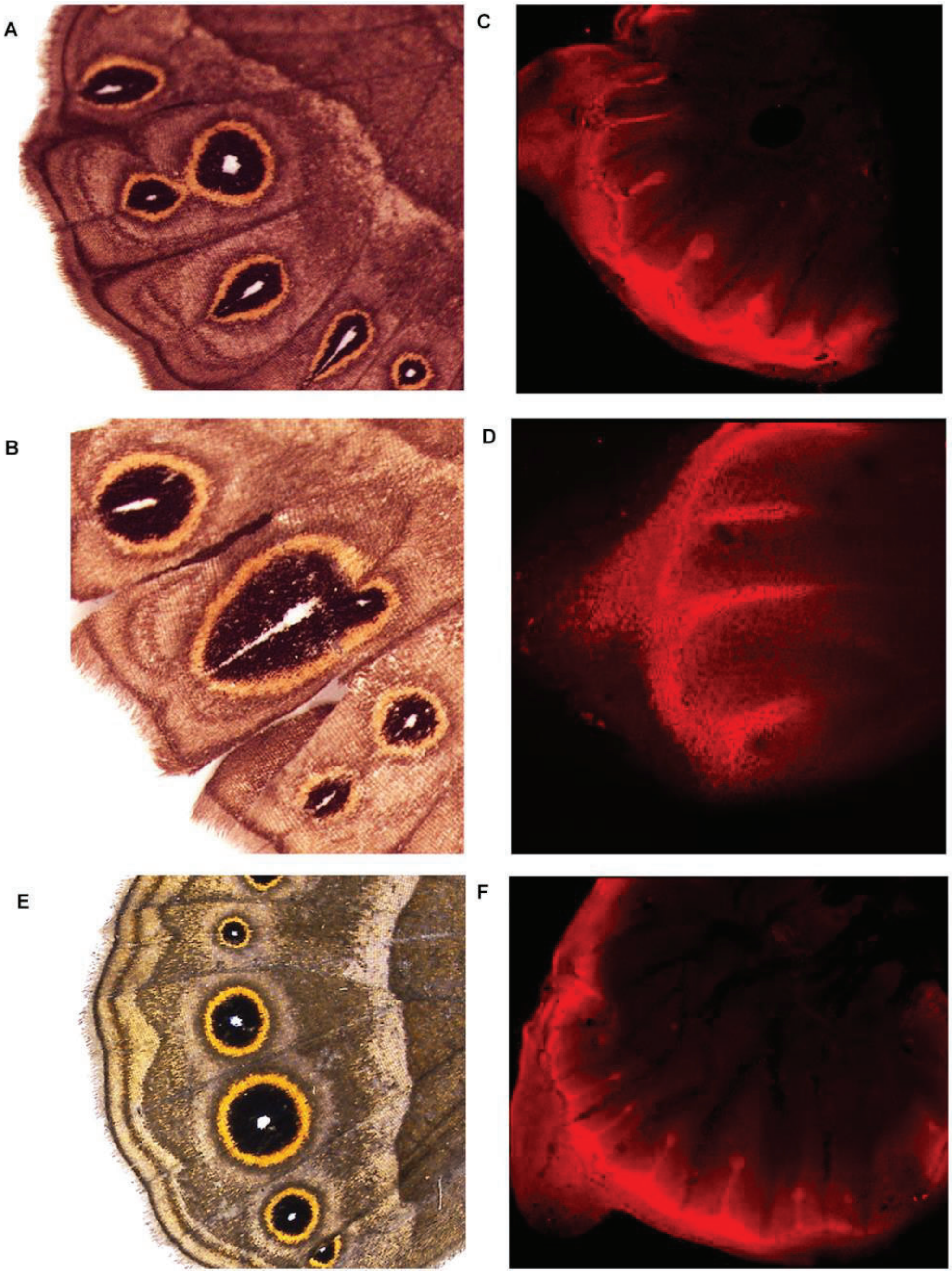

Development • Supplementary information

Fig. S11. (**A**) Phase diagram of $[A_1]$ field after 6 days of simulation with $\alpha = 6.2 \times 10^{-3}\ molecules.\mu m^{-2}.s^{-1}$, $K = 1.8 \times 10^{-7}\ molecules^{-2}.\mu m^{4}.s^{-1}$, $k_1 = 0.1 \times 10^{-3} s^{-1}$, $k_2 = 0.08 \times 10^{-3} s^{-1}$ depending of parameters D_1 and D_2. (**B**) More refined phase diagram around the parameters used in Fig. 3F.

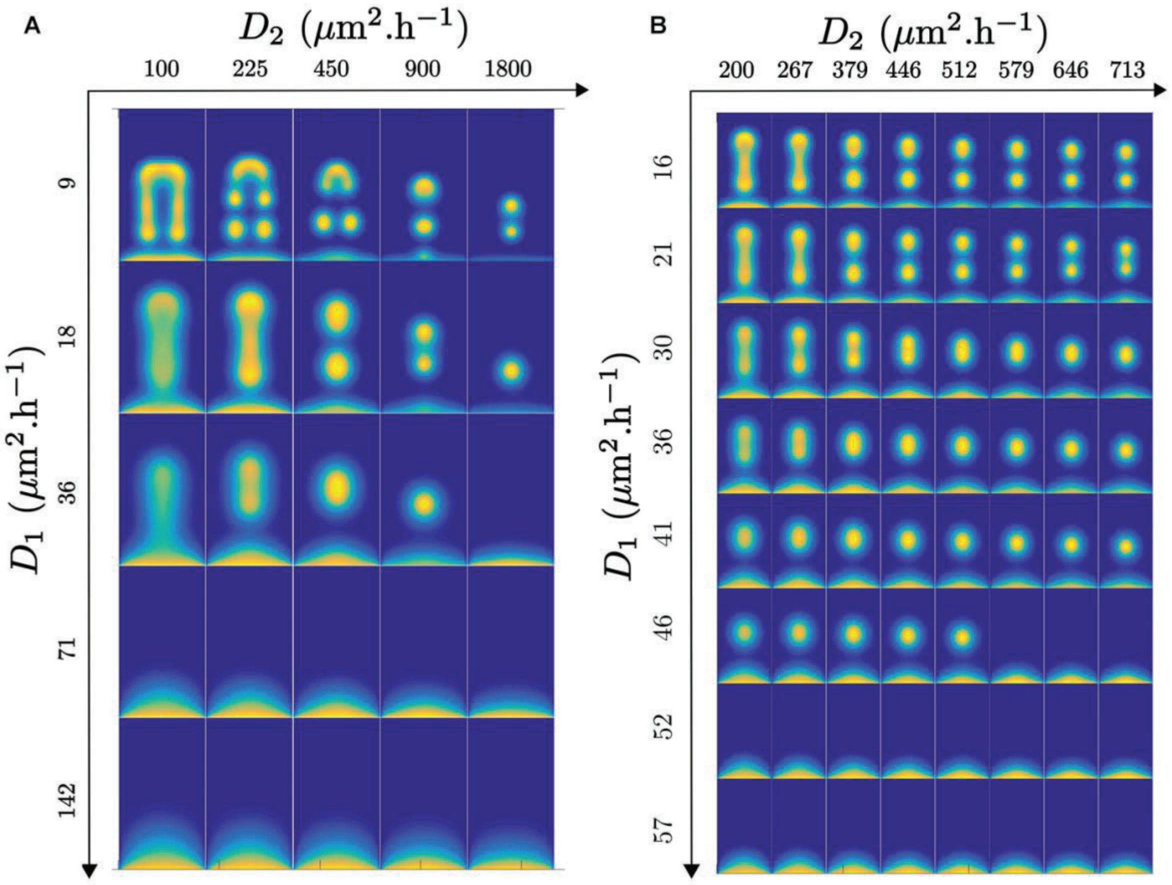

Development • Supplementary information

Fig. S12. Identification of exon 3 clones and wing cells and simplification of the clones and wing cells geometry. (**A,B**) Wild type forewing. (**C**) Wild type hindwing. (**D**) Exon 3 crispant forewing with one deformed eyespot and one missing eyespot. (**E**) Exon 3 crispant forewing with one deformed eyespot and one unaffected eyespot. (**F**) Exon 3 crispant hindwing with one missing eyespot, three unaffected eyespots and three splited eyespots. (**G,H,I**) : same as (**D,E,F**) with clones manually segmented and artificially colored in blue and wing cells contours manually segmented and contoured in green. (**J,K,L**) : same as (**G,H,I**) with the geometry of clones and wing cells simplified for the eyespots represented in Fig. 4B,C,E,F,G,H. The simplified clones geometry are in red and the simplified rectangular geometries of wing cells are in green.

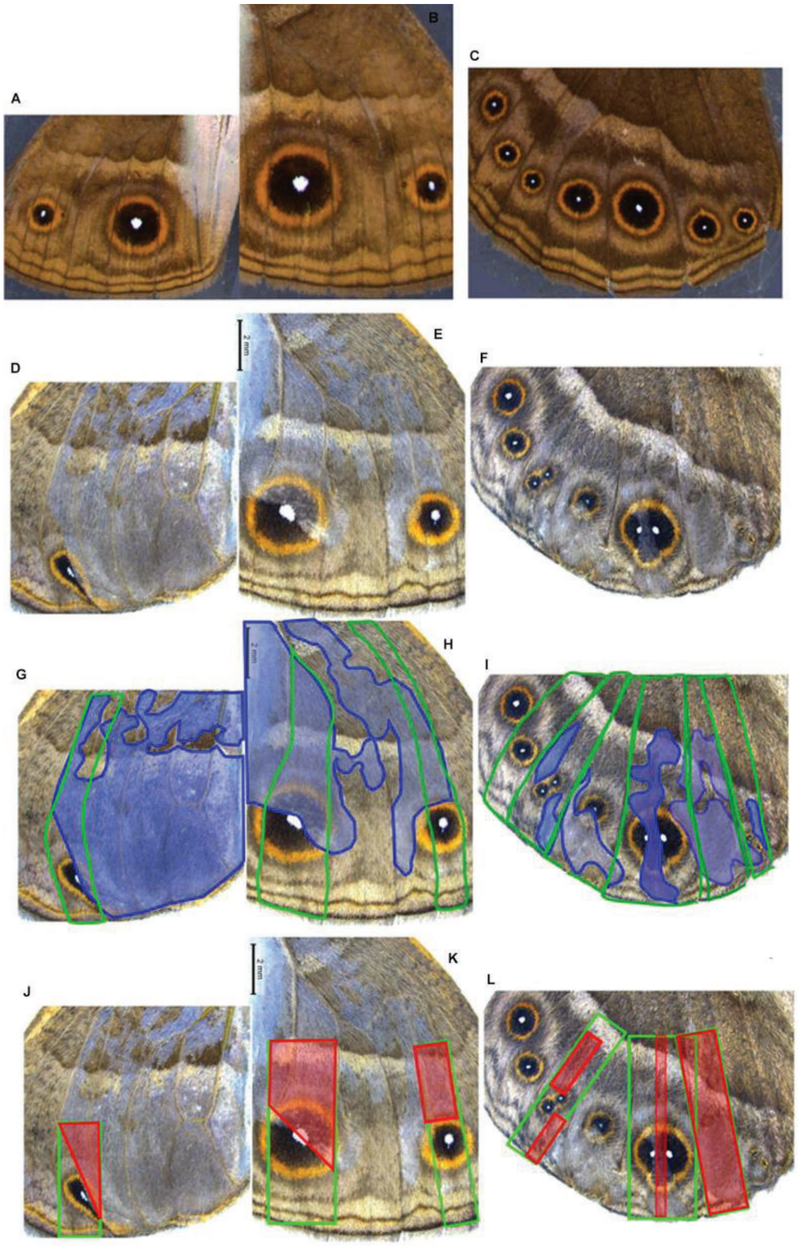

Development • Supplementary information

Fig. S13. Time-lapse results of reaction-diffusion using the same parameters than in Fig. 3F except that K = 0 in the clone region. Concentration of A_1 (first row) and A_2 (second row) over 6 days. Third row shows the overlap of A_1 and A_2. Fourth row represents square of Dll concentration. (**A**) Full clone (same as Fig. 4B). (**B**) Sliver clone (same as Fig. 4D). (**C**) Diagonal clone (same as Fig. 4E). (**D**) Comet clone (same as Fig. 4F). (**E**) Center clone (same as Fig. 4G). (**F**) Corner clone (same as Fig. 4H).

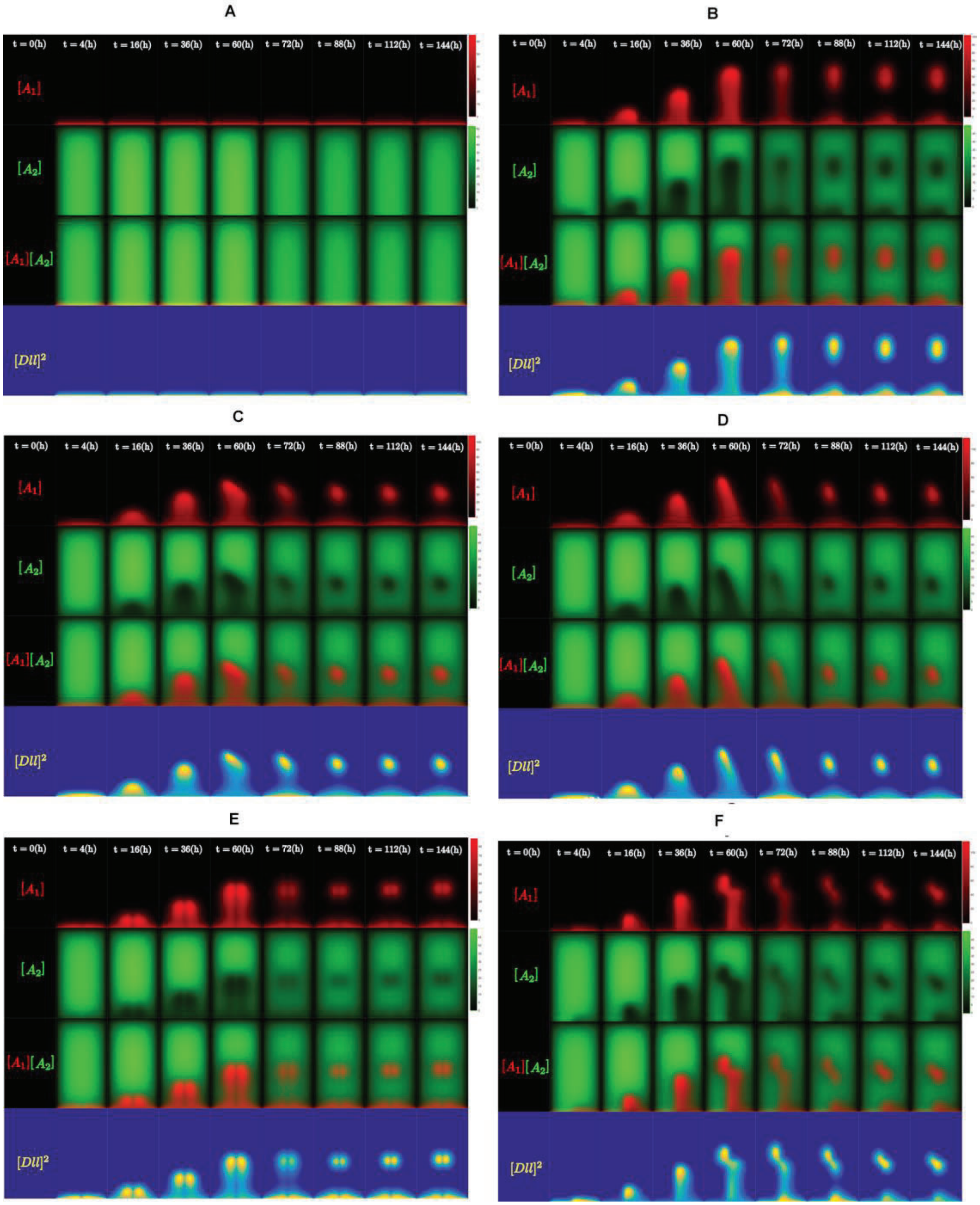

Development • Supplementary information

Fig. S14. Time-lapse results of reaction-diffusion simulation with same parameters than in Fig. 5C except that $K = 8.25 \times 10^{-4}\ molecules^{-2}.\mu m^{4}.h^{-1}$ instead of the wild-type case where $K = 6.5 \times 10^{-4}\ molecules^{-2}.\mu m^{4}.h^{-1}$. Concentration of A_1 (first row) and A_2 (second row) over 6 days. Third row shows the overlap of A_1 and A_2. Fourth row represents square of Dll concentration (which is the input into the system of equations described by Eq. 1-2) to see more clearly local maxima.

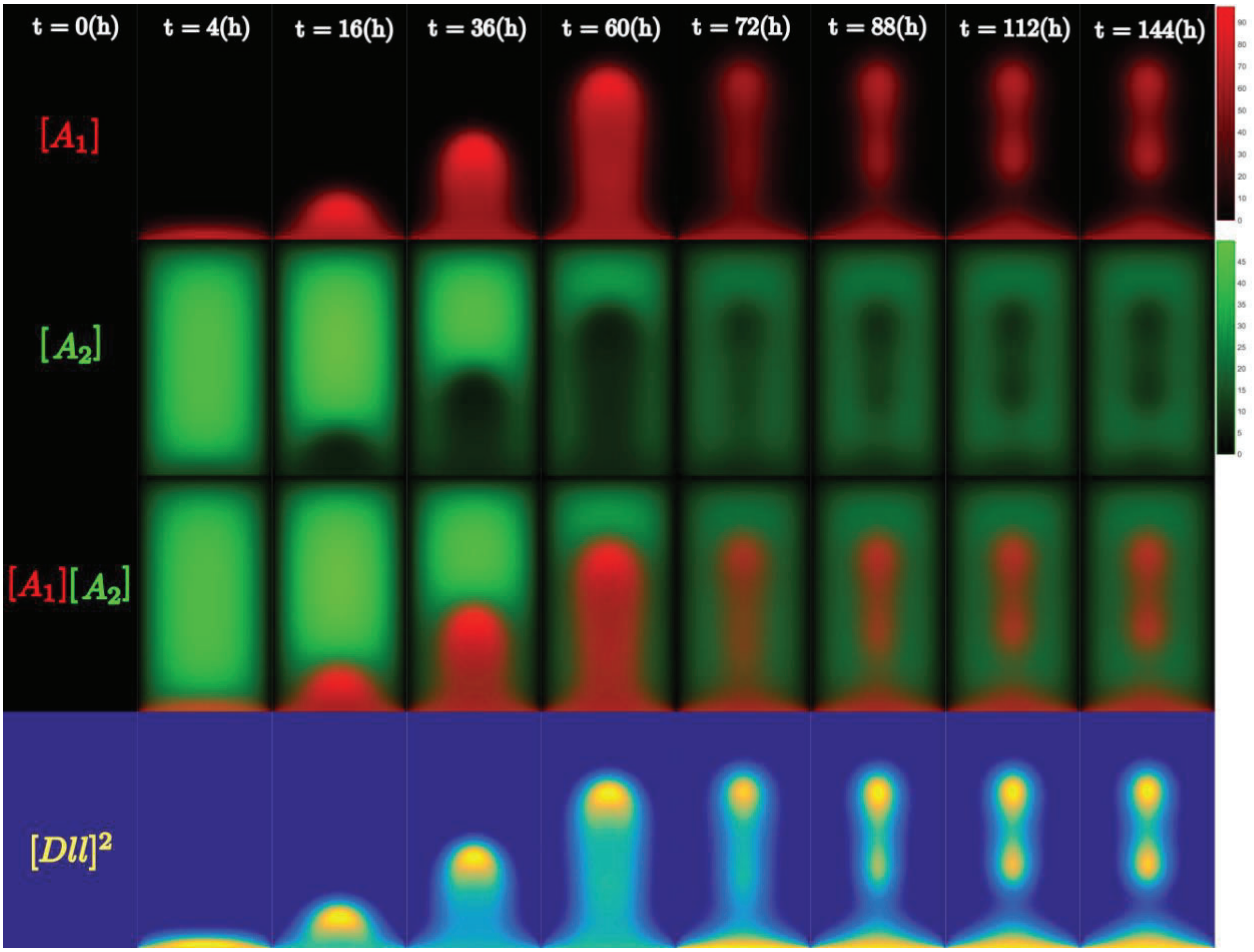

Fig. S15. (**A**) Boundary conditions imposed in the activator-inhibitor simulations. (**B**) Interaction network involving the activator A_1, the inhibitor A_2 and Dll. Dll interacts cooperatively with itself to induce A_1 and A_2. A_1 production is inhibited by A_2.

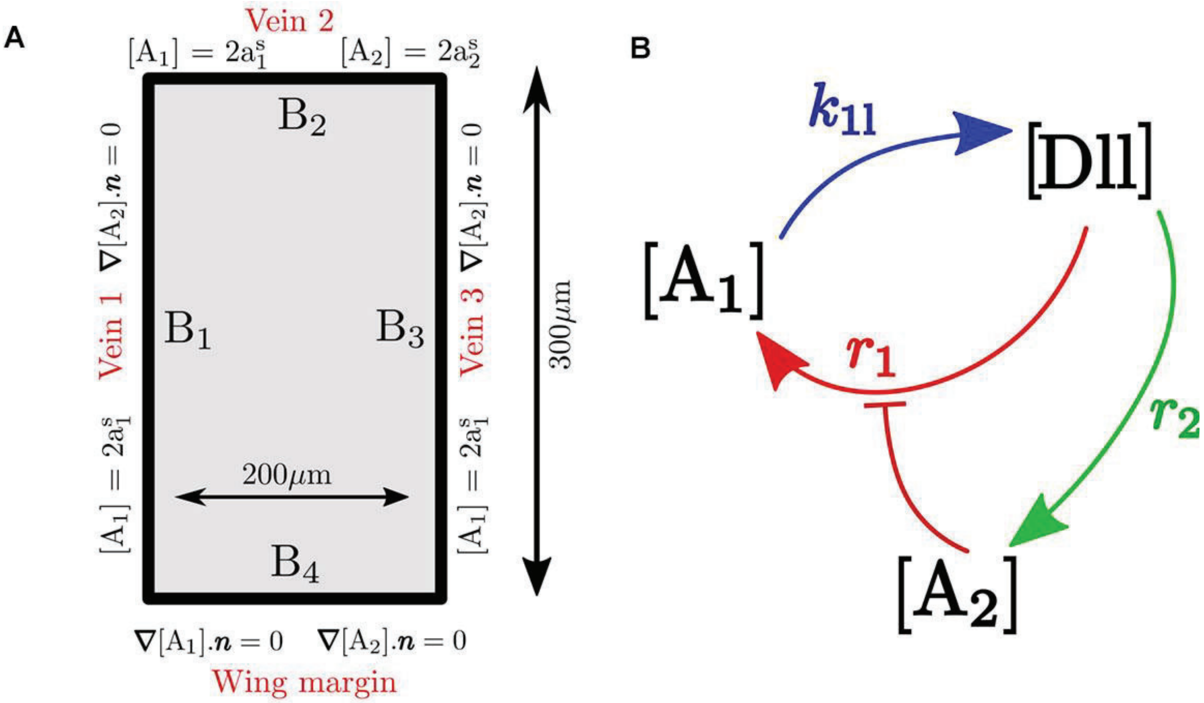

Development • Supplementary information

Fig. S16. Reaction-diffusion simulations of wing sectors where part of the sector (cyan outline) has no "activator" function. (**A**) Clones delimitation on the activator pattern at time t = 1h, t = 15h and t = 50h. (**B**) Time-lapse results of Geier-Meinhardt reaction-diffusion simulations with parameters in Table S7. Concentration of A_1 (first line) and A_2 (second line) over 50h (time t = 0h, 10h, 15h, 25h, 35h, 50h).

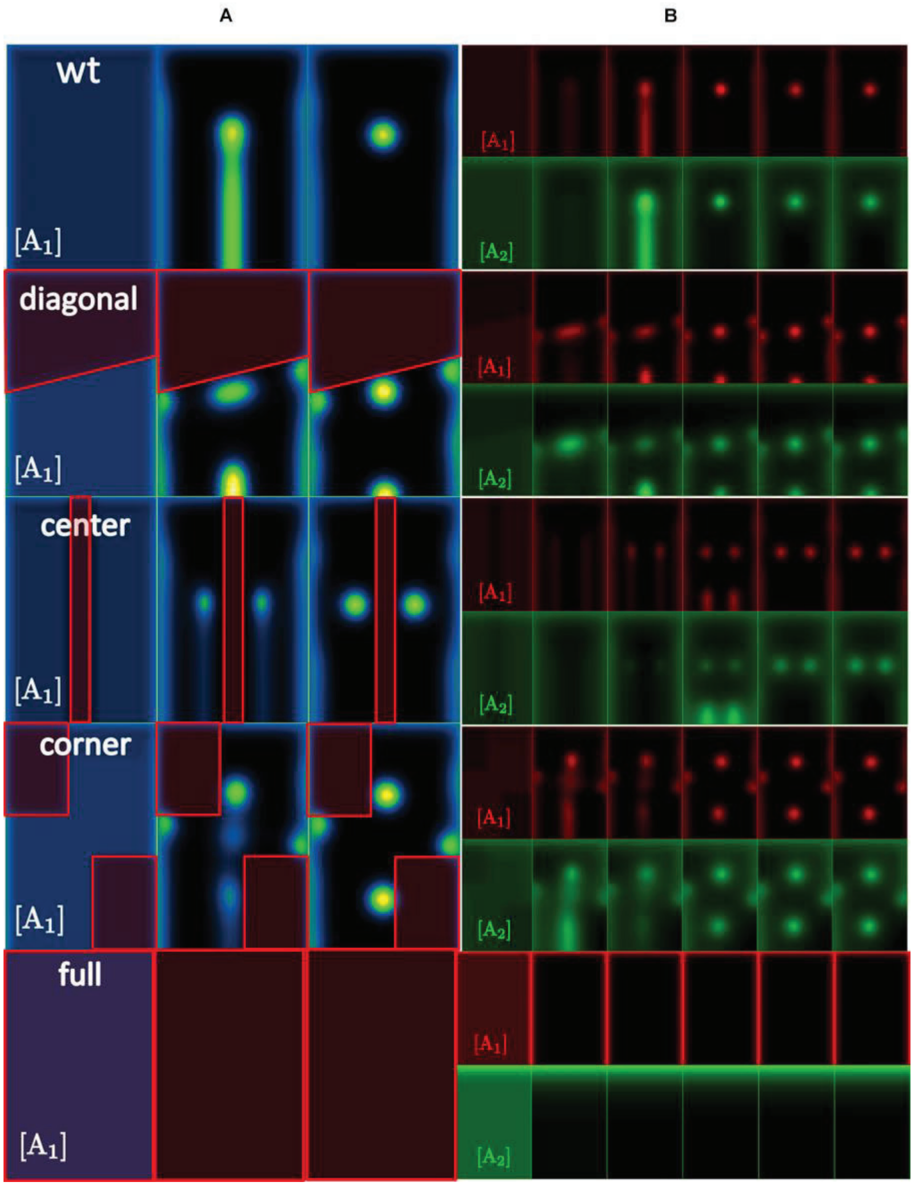

Development • Supplementary information

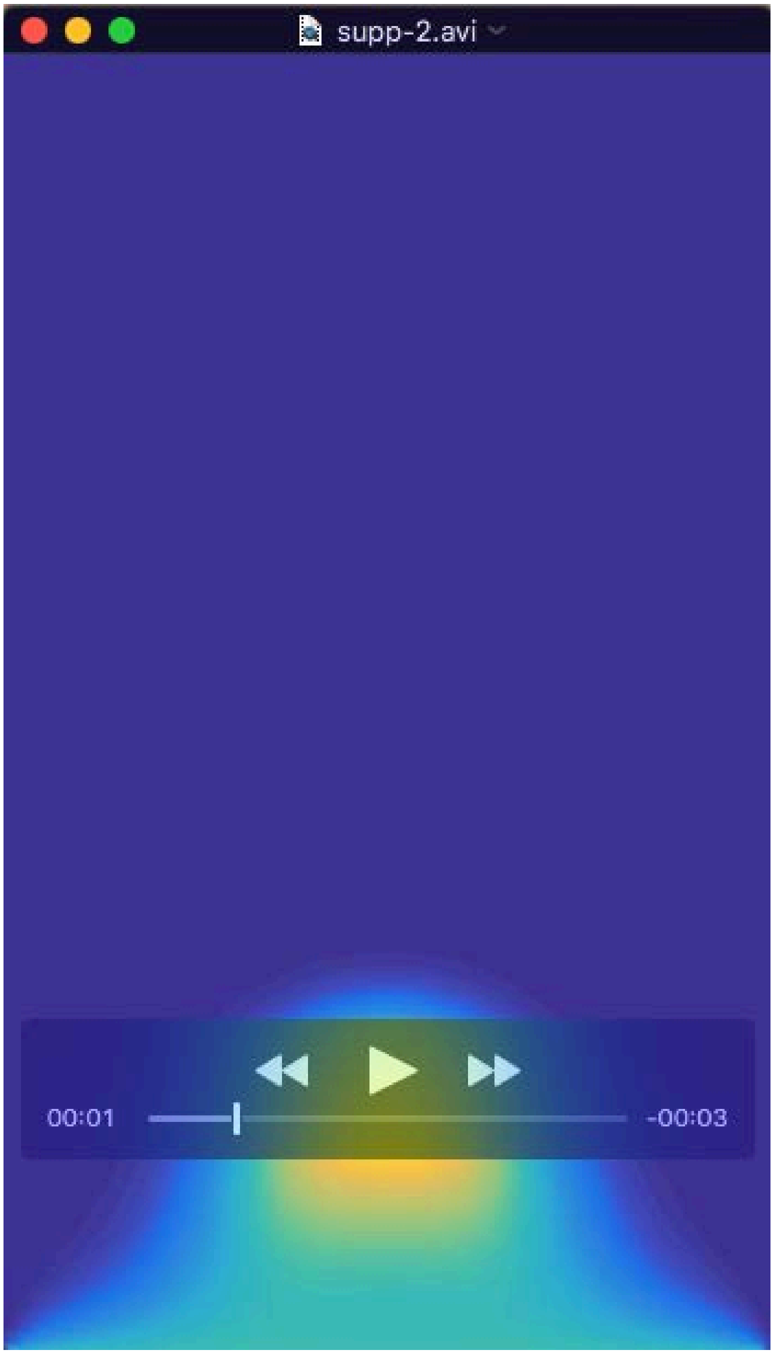

Movie 1 – Movie of Wild-type version of Gray-Scott model (same parameters than in Fig 3F)

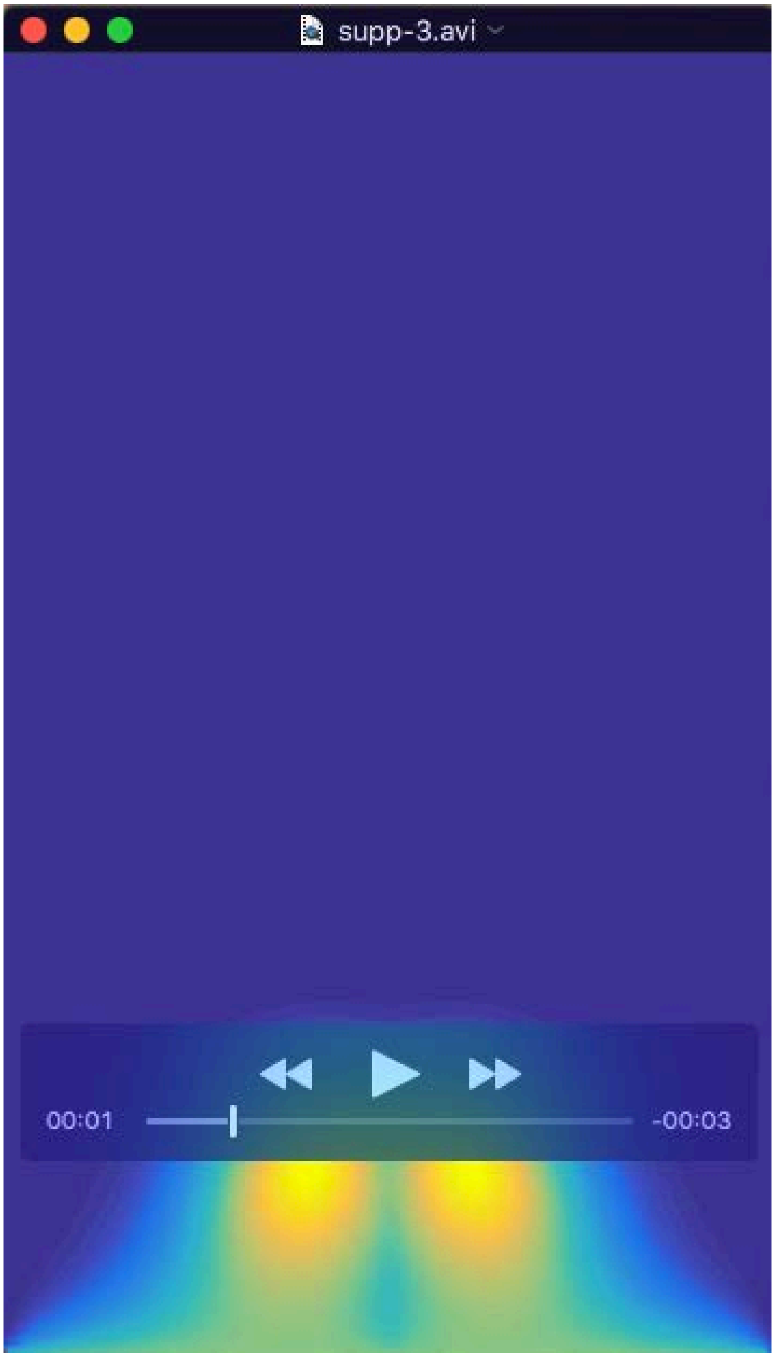

Movie 2 – Example movie of Gray-Scott model with the center mutant patch (same parameters than in Fig 4G).

Table S1 | Mapping statistics from CRISPResso next generation sequence analysis

Crispant	No. Input reads	No. Reads aligned to WT reference	Unmodified	Insertions	Deletions	Substitutions
Fig 2C	12163	11258	5968	418	4420	480
Fig 2D	13828	13402	10737	20	1879	806
Fig 2F	148827	141993	126890	1363	12174	1922
Fig 2G comet	149589	144345	12823	62124	52811	34110
Fig 2I Ectopic	120337	120020	34535	52716	27357	6010
Fig 2I Missing	236378	235860	41765	146875	35990	12152

Table S2 | Proportion of different mutations identified from the CRISPResso analysis of next generation sequencing from crispants obtained with Sg1 (5'UTR Exon 2) and Sg3 (Exon 3)

Crispant	Guide	Non-coding mutations %
Fig 2C	Sg3	0.0
Fig 2D	Sg3	0.0
Fig 2F	Sg1	96.6
Fig 2G Comet	Sg1	96.6
Fig 2I Ectopic	Sg1	95.7
Fig 2I Missing	Sg1	95.3

Development • Supplementary information

Table S3. Results from ESEfinder identifying 27 predicted exonic splice enhancers in exon 2 of Distal-less. The results are sorted by the highest scoring motifs. The top 3 motifs are underlined in the sequence of exon 2 below the table (*motifs for results 2 and 3 are identical). Results from ESEfinder show that the top scoring motifs are found in both guides Sg1 and Sg2 (red font). Sequence highlighted in blue = UTR, sequence in grey = coding region. Start site ATG is in bold font. Splicing is observed following injections of either Sg1 or Sg2.

Seq	Motif	Position	Site	Score
Exon2	SRSF1	127	CGGACGG	5.16885
Exon2	SRSF1	217	*CCCACGA	4.86363
Exon2	SRSF1	367	*CCCACGA	4.86363
Exon2	SRSF1	169	CCCAGGA	4.25952
Exon2	SRSF1	11	CGGCCGG	4.17531
Exon2	SRSF1	53	TACACGA	3.6386
Exon2	SRSF1	200	GGGAGGT	3.36097
Exon2	SRSF1	292	AACACGG	3.35712
Exon2	SRSF1	311	CAAAGGT	3.32172
Exon2	SRSF1	3	GCCACGA	3.28633
Exon2	SRSF1	138	GGCCCGT	3.21777
Exon2	SRSF1	436	CGTACGG	3.10507
Exon2	SRSF1	357	CAGCAGA	2.82558
Exon2	SRSF1	26	CGGAGCA	2.71378
Exon2	SRSF1	481	CGCCCAA	2.57058
Exon2	SRSF1	195	CACCTGG	2.34503
Exon2	SRSF1	167	CACCCAG	2.29102
Exon2	SRSF1	81	CCCACCA	2.2859
Exon2	SRSF1	249	CCCACCA	2.2859
Exon2	SRSF1	331	CCCACCA	2.2859
Exon2	SRSF1	12	GGCCGGG	2.24011
Exon2	SRSF1	253	CCAACGT	2.20038
Exon2	SRSF1	401	CTTAGGT	2.19919
Exon2	SRSF1	141	CCGTCGA	2.16653
Exon2	SRSF1	90	CTGAGTT	2.14295
Exon2	SRSF1	153	CAGCCCG	2.04479
Exon2	SRSF1	344	CAGCCCG	2.04479

GCGCCACAAGCGGCCGGGTAGCGTTCGGAGCAGTCCAGAAATCCCTCAAAATTACACGAATTCAGTCCCCAAACA
GCAAACCCACCACGCTGAGTTTCTCAGATCCCTTTGGGCCTCCTCAGTCCACGGACGGGGGGGGCCCGTCGACCC
CGCAGCCCGCC**ATG**ACCACCCAGGAGCTGGACCACCAACACCACCACCTGGGAGGTTCCCAAACCCCCCACGAT
ATATCCAACTCCACGAATTCCACCCCCACCAACGTTTCCTCGAAATCCGCCTTCATAGAGTTACAGCAACACGGT
TACGGGCCTTTCAAAGGTGGTTACCAACATCCCCACCATTTCGGCAGCCCGGGGGGTCAGCAGAACCCCCACGAG
GCGTCGGGGTTCCCGAGCCCTAGGTCCTTAGGTTATCCCTTTCCTCCTATGCACCAAAATACGTACGGATATCAC
ATAGGCTCCTACGCTCCACAATGCGCAAGTCCGCCCAAAGATG

Development • Supplementary information

Table S4. Primers and sgRNA sequences used in this study

**Primers:**	**DNA sequences (5' to 3')**
Dll exon 2 forward (*in vitro* assay)	CGGAGACCGTCAGTTATTTTGA
Dll exon 2 reverse (*in vitro* assay)	TGGATCCCATGGATGAGTCA
Dll exon 3 forward (*in vitro* assay)	TCAGTGACTGGTTCCCTTGC
Dll exon 3 reverse (*in vitro* assay)	AATAGTCCACGCCTTGCTT
Dll exon 2 forward (Illumina)	GCAGTCCAGAAATCCCTCAA
Dll exon 2 forward (Illumina)	TTGCACATTGAGGAGCGTAG
Dll 1.5kb product (cDNA)	CGTACGCCGATTTCGTTCGGAC
Dll 1.5kb product (cDNA)	GCTTCACATCACAGGAGGTGCC
Dll exon 1 forward (qPCR)	TGAGGTGATAGCCGAATCGC
Dll exon 1 reverse (qPCR)	ATTGACGGCAATTAGCGGGA
EF1 alpha forward (qPCR)	GTGGGCGTCAACAAAATGGA
EF1 alpha reverse (qPCR)	GCAAAAACAACGAT
Dll Exon2 5'UTR guide	GAAATTAATACGACTCACTATAGGGCCTCCTCAGTCCACGGAGTTTTAGAGCTAGAAATAGC
Dll Exon2 cds guide	GAAATTAATACGACTCACTATAGGGTCAGCAGAACCCCCACGGTTTTAGAGCTAGAAATAGC
Dll Exon3 guide (reverse strand)	GAAATTAATACGACTCACTATAGGAAGCTGGAGTAGATGGTGCGGTTTTAGAGCTAGAAATAGC
Common reverse guide	AAAAGCACCGACTCGGTGCCACTTTTTCAAGTTGATAACGGACTAGCCTTATTTTAACTTGCTATT TCTAGCTCTAAAAC
**SgRNA guide targets: 5'-3'**	(PAM sequence in red)
Dll SgRNA 1 Exon 2	GGGCCTCCTCAGTCCACGGACGG
Dll SgRNA 2 Exon 2	GGGTCAGCAGAACCCCCACGAGG
Dll SgRNA Exon 3	CCGCGCACCATCTACTCCAGCTT
***in situ* probes**	
Dpp Forward	GTTCTTCAACGTAAGCGGCG
Dpp Reverse	CCACAGCCTACCACCATCAT

*Dll Exon 3 primers used for next generation sequencing were the same as those used for the cleavage assay with the addition of a 5 bp barcode on the 5' end of each primer.

Development • Supplementary information

Table S5 | Measured dynamic parameters of Wg and Dpp in *Drosophila*. Parameters extracted from Kicheva et al., (2007).

	L	T_{dev}	D_{wg}	D_{Dpp}	k_{wg}	k_{dpp}	α_{wg}	α_{wg}	α_{dpp}	α_{dpp}
Value	100	1	0.04	0.1	1.4×10^{-3}	0.2×10^{-3}	20	200×10^{-3}	2	20×10^{-3}
Unit	μm	days	$\mu m^2/s$	$\mu m^2/s$	s^{-1}	s^{-1}	$molecules .cell^{-1}.s^{-1}$	$molecules .\mu m^{-2}.s^{-1}$	$molecules .cell^{-1}.s^{-1}$	$molecules .\mu m^{-2}.s^{-1}$

Table S6. Parameters used for the simulations presented in Figs. 3,4, Fig. S11.

	L_x	L_y	T_{simu}	D_1	D_2	k_1	k_2	K	α	α_{late}	T_{late}	c_{margin}
Value	150	262	6	0.01	0.12	0.1×10^{-3}	0.08×10^{-3}	1.8×10^{-7}	6.2×10^{-3}	$0.75 * \alpha$	2.5	65
Unit	μm	μm	days	$\mu m^2/s$	$\mu m^2/s$	s^{-1}	s^{-1}	$molecules^{-2} .\mu m^4.s^{-1}$	$molecules .\mu m^{-2}.s^{-1}$	$molecules .\mu m^{-2}.s^{-1}$	days	$molecules .\mu m^{-2}$

Table S7. Parameters used for the simulations presented in Figs. S15,16.

	L_x	L_y	T_{simu}	D_1	D_2	k_1	k_2	K_1	K_2
Value	200	300	50	0.009	0.083	0.17×10^{-3}	0.07×10^{-3}	0.17×10^{-3}	0.17×10^{-3}
Unit	μm	μm	hours	$\mu m^2/s$	$\mu m^2/s$	s^{-1}	s^{-1}	s^{-1}	$molecules^{-1} .\mu m^2.s^{-1}$

Development • Supplementary information

Supplementary Materials and Methods

Theoretical modeling

The Gray-Scott Model Equations

The system for eyespot formation is defined by three coupled equations in two spatial dimensions:

$$\frac{\partial[A_1]}{\partial t} = r_1([Dll]^2[A_2]) - k_1[A_1] + D_1(\frac{\partial^2[A_1]}{\partial x^2} + \frac{\partial^2[A_1]}{\partial y^2})$$

$$\frac{\partial[A_2]}{\partial t} = \alpha - r_2([Dll]^2[A_2]) - k_2[A_2] + D_2(\frac{\partial^2[A_2]}{\partial x^2} + \frac{\partial^2[A_2]}{\partial y^2})$$

$$\frac{\partial[Dll]}{\partial t} = k_{1l}[A_1] - k_3[Dll]$$

The first equation describes A_1 dynamics, which diffuses with a diffusion coefficient D_1, is degraded at a rate k_1 and produced at a rate $r_1([Dll]^2[A_2])$ which means that A_1 production is catalyzed cooperatively by Dll and A_2.The second equation describes A_2 dynamics, which diffuses with a diffusion coefficient D_2, is degraded at a rate k_2 and produced at a homogenous rate α. The term $-r_2([Dll]^2[A_2])$ can be interpreted as a Dll dependant degradation rate of A_2 or as a Dll and A_2 dependant modulation of A_2 production. A_1 and A_2 likely represent complex interaction motifs, so the constants (degradation, diffusion) represent *effective* parameters and may not reflect the precise molecular dynamics.

In the following we assumed that the system is tuned to equalize r_1 and r_2 such that the system stays within the parameter region of the Gray-Scott model that leads to spot formation. This assumption corresponds to a detailed balance approximation implying that A_2 is degraded during A_1 production. As described in the previous paragraph, this could correspond to a real A_2 degradation or to A_2 downregulation.

The last equation describes the dynamics of Dll which is activated by A_1 at a rate $k_{1l}A_1$ and degraded at a rate k_3. Dll concentration $[Dll]$ follows the concentration $\frac{k_{1l}[A_1]}{k_3}$ with a characteristic delay time $\tau = \frac{1}{k_3}$. In the following, we took a quasi-static assumption for Dll dynamics, assuming that the degradation time τ is much shorter than the other characteristic timescales of the system (timescales of diffusion on relevant length scales and degradation of the morphogens). In the following $[Dll]$ is taken as proportional to $[A_1]$ which is in agreement with Dll and armadillo co-localization (Fig. 3B,C).

Integrating these last assumptions, the above equations become:

$$[Dll] = \frac{k_{1l}[A_1]}{k_3}$$

$$\frac{\partial[A_1]}{\partial t} = \frac{r_2 \times {k_{1l}}^2}{{k_3}^2} \times [A]_1{}^2 \times [A_2] - k_1[A_1] + D_1\Delta[A_1]$$

$$\frac{\partial[A_2]}{\partial t} = \alpha - \frac{r_2 \times {k_{1l}}^2}{{k_3}^2} \times [A]_1{}^2 \times [A_2] - k_2[A_2] + D_2\Delta[A_2]$$

which lead to the Gray Scott equations used in the main text with $K = \frac{r_2 \times {k_{1l}}^2}{{k_3}^2}$:

Development • Supplementary information

$$\frac{\partial[A_1]}{\partial t} = K[A_1]^2[A_2] \, - k_1[A_1] + \, D_1\nabla^2[A_1]$$

$$\frac{\partial[A_2]}{\partial t} = \alpha - K[A_1]^2[A_2] - k_2[A_2] + \, D_2\nabla^2[A_2]$$

Simulations

Simulations of A_1 and A_2 dynamics in the wing cell were implemented in MATLAB (MATLAB and Statistics Toolbox Release 2015b). The differential equations were rewritten numerically using the forward Euler integration of the finite-difference equations resulting from the discretization of the diffusion terms. The spatial mesh was a grid of 60×105 points which corresponds to a 2.5 μm meshsize. The time step used was of 0.0005 hours. The results presented were confirmed by changing grid size and time step, resulting in no qualitative change in simulation results.

Boundary conditions: Boundaries B_1 B_2 B_3, as shown in Fig. 3d, correspond to the wing-cell to wing-cell boundaries, delimitated by veins. The boundary B_4 corresponds to the wing-cell margin. [A_1] is imposed as constant along B_4 to account for the constant presence of wingless at the margin. A_2 flux normal to B_4 is imposed as null. To account for the absence of Dpp along the veins (Fig. S7), we impose a null concentration [A_2] along B_1, B_2, B_3 assuming the veins act as concentration sinks. We also impose a null concentration [A_1] along B_1, B_2, B_3.

$[A_1] = 0$ on B_1 B_2 B_3, $[A_1] = c_{margin}$ on B_4 .

$[A_2] = 0$ on B_1 B_2 B_3, $\frac{\partial[A_2]}{\partial y} = 0$ on B_4.

Parameter Estimation: Parameters values are motivated from direct measurements of Wg and Dpp dynamics in the *Drosophila* wing disc, (Kicheva et al., 2007)**.** D_1 is the diffusion coefficient of A_1 (putatively Wg) and D_2 is the effective diffusion coefficient of A_2 (putatively Dpp). In Kicheva et al., (2007), values of the effective diffusion coefficients of Wg and Dpp were experimentally measured in the developing fly wing using FRAP. Their degradation rate k_{wg} and k_{dpp} and production rates α_{wg} and α_{dpp} were also measured (See Table S4). Of course, these estimates are likely not exact for our system because (1) the butterfly wing-disc is significantly larger and (2) we are modeling effective interaction motifs, not single molecules. However, these estimates help to constrain the parameter and, as shown, the model is able to replicate a wide variety of phenotypes.

Phase diagram in the Gray-Scott model

The phase diagram of the Gray-Scott system is rich and the number of parameters to be explored are numerous. We explored first the different patterns that could emerge varying systematically degradation rates k_1, k_2, diffusion rates D_1, D_2, production rate α, reaction rate K and c_{margin} concentration around values estimated in Kicheva et al., (2007). A rich phase diagram was found as we could generate different types of patterns going from a v-shape pattern on the margin to a double spot pattern (see Fig. S8-9, S11).

Noticing that the single spot pattern was rarely formed as it was an intermediate state between the V-shape state and the finger state, we looked for ways to first generate a finger and stabilize it as a single spot after a certain time. As a dpp decrease of intensity was noticed in late larval stage (Fig. S7), we decreased the level of Dpp production α to α_{late} at time T_{late} to see the impact on the patterning. Decreasing α after finger emergence enabled to generate stable spot more frequently, though the single spot state was still a transient pattern in the transition zone between the “no spot state” and the “finger state” (Fig. S9).

Development • Supplementary information

We found through parameter exploration parameters that could reproduce the spatio-temporal dynamics of wild-type spot formation (Fig. 3) as well as most aspects of Dll clones phenotypes (Figs. 4,5). We explored the phase diagram around these optimal values, investigating first α and K variations with other parameters fixed (Fig. 5A, Fig. S9A) and then exploring D_1 and D_2 with other parameters fixed (Fig. S11). The parameters used in our simulation are shown in Table S5. The degradation rates, diffusion rates and production rates are in overall smaller than the corresponding parameters in *Drosophila,* reflecting the longer time scales involved in butterfly eyespot formation than in wing disk development in *Drosophila*.

α-K phase diagram: For low α and K, no pattern is obtained. At fixed α, spots start to appear above a critical K. Increasing K further leads to the transformation of the spot pattern in a double spot vertical pattern and then further to a finger pattern (Fig.5, Fig. S9A). More precisely, the spot pattern turns into a double spot pattern by progressively increasing its size up to a critical size were the spot splits in two spots (Fig. 5B). Increasing more K leads to a comet phenotype (Fig. 9A).

For Dll*,* k_{1l} is the production rate and k_3 its degradation rate. As $K = \frac{r_2 \times {k_{1l}}^2}{{k_3}^2}$, a decrease of Dll degradation rate leads to K increase, which could explain the vertical splitting spot phenotype. In Fig. S14**,** we show spot vertical splitting dynamics.

Geier-Meinhardt Model, developed from Sekimura et al. (2015).

Before developing our Gray-Scott equations based model, we first had investigated an adaptation of the Gierer-Meinhardt model developed in (Sekimura et al., 2015) including *Dll* as part of the activator loop. These simulations reproduced less accurately our experimental results than the Gray-Scott model. However, some characteristics – such as eyespot splitting - were still observed in this framework. This leads to the conclusion that *Dll* belonging to part of an activator loop in eyespot patterning is robust to changes in the specific reaction-diffusion models. In the following, we briefly describe the results of these simulations on the Geier-Meinhardt model.

The Gierer-Meinhardt model describes a reaction-diffusion system in which a short-range autocatalytic activator also activates its long-range antagonist, the inhibitor. Here, we adapted this model to include Dll in the same way as described above in the Gray Scott model, (Figs. S15,16). This system is defined by three coupled equations in two spatial dimensions:

$$\frac{\partial[A_1]}{\partial t} = r_1 \frac{[Dll]^2}{[A_2]} - k_1[A_1] + D_1(\frac{\partial^2[A_1]}{\partial x^2} + \frac{\partial^2[A_1]}{\partial y^2})$$

$$\frac{\partial[A_2]}{\partial t} = r_2([Dll]^2) - k_2[A_2] + D_2(\frac{\partial^2[A_2]}{\partial x^2} + \frac{\partial^2[A_2]}{\partial y^2})$$

$$\frac{\partial[Dll]}{\partial t} = k_{1l}[A_1] - k_3[Dll]$$

The first equation describes A_1 dynamics, which diffuses with a diffusion coefficient D_1, is degraded at a rate k_1 and produced at a rate $r_1 \frac{[Dll]^2}{[A_2]}$ which means that A_1 production is catalyzed by Dll and inhibited by A_2. The second equation describes A_2 dynamics, which diffuses with a diffusion coefficient D_2, is degraded at a rate k_2 and produced at a rate $r_2([Dll]^2)$. Taking the same quasistatic assumption on Dll dynamics as in the Gray-Scott model, we assume that

$$[Dll] = \frac{k_{1l}[A_1]}{k_3}$$

This leads to the equations

$$\frac{\partial [A_1]}{\partial t} = K_1 \frac{[A_1]^2}{[A_2]} - k_1[A_1] + D_1 \nabla^2 [A_1]$$

$$\frac{\partial [A_2]}{\partial t} = K_2 [A_1]^2 - k_2[A_2] + D_2 \nabla^2 [A_2]$$

with $K_1 = \frac{r_1 \times {k_{1l}}^2}{{k_3}^2}$ and $K_2 = \frac{r_2 \times {k_{1l}}^2}{{k_3}^2}$.

The system has a steady state ($[A_1] = a_1^s = \frac{K_1 k_2}{K_2 k_1}$, $[A_2] = a_2^s = \frac{{K_1}^2 k_2}{K_2 {k_1}^2}$).

Parameters values for the simulations presented in the Table S6.

References

Akiyama, T. and Gibson, M. C. (2015). Decapentaplegic and growth control in the developing Drosophila wing. *Nature* **527**, 375–8.

Kicheva, A., Pantazis, P., Bollenbach, T., Kalaidzidis, Y., Bittig, T., Jülicher, F. and González-Gaitán, M. (2007). Kinetics of Morphogen. *Science* **315**, 521–526.

Monteiro, A., Prijs, J., Bax, M., Hakkaart, T. and Brakefield, P. M. (2003). Mutants highlight the modular control of butterfly eyespot patterns. *Evol. Dev.* **5**, 180–7.

Reed, R. D., Chen, P.-H. and Frederik Nijhout, H. (2007). Cryptic variation in butterfly eyespot development: the importance of sample size in gene expression studies. *Evol. Dev.* **9**, 2–9.

Sekimura, T., Venkataraman, C. and Madzvamuse, A. (2015). A model for selection of eyespots on butterfly wings. *PLoS One* **10**, 1–24.
